# Oncolytic Therapy: Delivery System and New Therapeutic Strategies for Cancer

**DOI:** 10.1002/mco2.70725

**Published:** 2026-04-09

**Authors:** Sikan Jin, Yi Zhang, Ziling Zhou, Yaqi Zhang, Ting Yu, Rui Xu, Lin Xu, Jidong Zhang, Longze Zhang, Shan Yang, Xianyao Wang

**Affiliations:** ^1^ Department of Immunology & Key Laboratory of Cancer Prevention and Treatment of Guizhou Province Zunyi Medical University Zunyi China; ^2^ Scientific Research Center The Third Affiliated Hospital of Zunyi Medical University Zunyi China; ^3^ Department of Genetics Zunyi Medical University Zunyi China

**Keywords:** antitumor therapy, combination treatment strategies, immunotherapy, oncolytic viruses, viral delivery

## Abstract

Oncolytic virotherapy is an emerging cancer immunotherapy that combines selective tumor cell lysis with activation of systemic antitumor immunity. Various DNA‐ and RNA‐based oncolytic viruses (OVs) have demonstrated favorable safety profiles and therapeutic activity across different malignancies. Despite these advancements, clinical efficacy remains inconsistent because of several biological barriers, including rapid immune clearance, insufficient tumor targeting, limited intratumoral spread, and the immunosuppressive tumor microenvironment (TME). In this review, we examine the key mechanisms of OV infection, tumor selectivity, and virus‐induced antitumor immune responses. It also explores the factors that limit therapeutic efficacy, particularly host antiviral immunity, structural barriers within solid tumors, and the immunosuppressive networks in the TME. To address these challenges, a range of strategies have been developed, with a focus on optimizing viral delivery. Current approaches, such as cell‐based carriers, extracellular vesicle‐mediated transport, and nanomaterial‐assisted delivery systems, aim to enhance tumor targeting, protect viral integrity, and improve intratumoral distribution. Additionally, combination therapies designed to enhance antitumor immunity and reshape the TME are outlined, including immune checkpoint blockade, chemoradiotherapy, and metabolic modulation. Collectively, these advancements transform OVs from standalone cytolytic agents into adaptable immunotherapeutic platforms, with their effectiveness determined by the delivery method, microenvironmental conditions, and therapeutic integration.

## Graphical abstract

This review summarizes recent advances in nanoparticle‐ and cell‐mediated OVs delivery strategies that improve biodistribution and tumor targeting while limiting immune clearance. It also examines combination approaches that enhance therapeutic efficacy through tumor microenvironment modulation or immune activation, thereby overcoming immunosuppression and strengthening antitumor immunity.

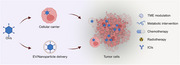

## 1 Introduction

Cancer continues to be a leading cause of global disease burden. Projections indicate that rates will rise significantly over the next two decades, posing a severe threat to public health and development [1]. The growing incidence and mortality rates highlight the urgent need for more effective treatment strategies. Although conventional modalities such as surgery, chemotherapy, radiotherapy, and targeted therapy have improved patient outcomes, they are often limited by adverse effects and their suboptimal efficacy against certain malignancies.

With a deeper understanding of the tumor immune microenvironment, immunotherapy has emerged as a transformative advancement in oncology. By harnessing the immune system to target and eliminate malignant cells, it has revolutionized cancer treatment and reinvigorated the field of tumor immunology. Accumulating evidence suggests that immune cell infiltration within the tumor microenvironment (TME) plays a critical role in tumor progression and is closely associated with patient prognosis [2]. Various immunotherapeutic strategies, including immune checkpoint inhibitors (ICIs), cancer vaccines, adoptive cell transfer, and oncolytic virotherapy (OVT), have demonstrated promising clinical outcomes and continue to highlight the clinical value of immunotherapy in cancer management [3]. Among these immunotherapeutic strategies, OVT has attracted increasing attention as an emerging cancer treatment because of its dual ability to lyse tumor cells directly and stimulate antitumor immune responses. Oncolytic viruses (OVs) selectively infect and destroy malignant cells, while simultaneously promoting immune activation through the release of tumor‐associated antigens (TAAs), induction of inflammatory responses, and modulation of the TME [4, 5, 6]. These features offer a novel and promising approach for enhancing the overall effectiveness of cancer immunotherapy.

As early as the mid‐20th century, clinicians have documented occasional cases of tumor regression occurring alongside natural viral infections [7]. Since then, a diverse array of OVs has been developed and tested against various cancer types. Despite their different origins, these viruses share a common ability to selectively infect and lyse malignant cells, often inducing immunogenic cell death (ICD) that converts “cold” tumors into “hot” tumors by promoting tumor antigen release and immune activation [7]. Extensive preclinical studies and an increasing number of clinical trials have demonstrated that OVs can be delivered safely, with certain patients achieving profound and durable tumor responses [4]. The translational impact of OVT has been highlighted in several recent regulatory approvals worldwide. In 2005, an oncolytic adenovirus (H101) was approved for use in China. A decade later, the herpes simplex virus (HSV)‐based talimogene laherparepvec was approved in the United States and European Union. Additionally, in 2021, a third‐generation HSV construct (G47Δ) was approved in Japan for the treatment of glioblastoma [7]. Together, these milestones highlight the vast therapeutic potential of OVT across various viruses and clinical contexts.

Despite this progress, significant clinical challenges continue to limit the broader implementation of OVT [4]. A central obstacle lies in the complex host–tumor interface that OVs must navigate, where solid tumors often impose physical and immunological barriers, such as abnormal vasculature, dense stroma, and immunosuppressive microenvironments, that restrict viral spread and replication. In addition to this complexity, the host immune system presents a dual challenge. While OVs are engineered to stimulate antitumor immunity, preexisting antiviral defenses, including neutralizing antibodies or rapid innate immune responses, can clear systemically administered viruses before they reach the tumor site [8]. This is one reason why many trials have relied on intratumoral OV administration. Furthermore, achieving an optimal balance between viral safety and therapeutic efficacy remains challenging, as attenuation for clinical use often compromises the replication capacity and oncolytic efficacy of the viruses within tumors [9]. Collectively, these challenges explain why OVT has not yet become a mainstream treatment and highlight the scientific hurdles that ongoing research must address.

Here, we synthesized the results from major OV classes and provided an updated overview of the OVT landscape, offering insights into how emerging innovations may address current limitations. Our review begins with a concise historical context and outlines the fundamental mechanisms by which OVs infect and kill tumor cells, including a classification of key viral families. We subsequently examined the principal barriers that constrain OVT efficacy in the clinic and explored novel strategies to overcome them. Next, we discussed advanced delivery strategies such as cell‐based carriers, vesicle‐based vehicles, and nanotechnology‐enabled systems, which enhance tumor targeting and viral persistence. We also surveyed rational combination approaches designed to remodel the TME and potentiate antitumor immunity, thereby amplifying the effects of OVT. Finally, we summarized the current clinical trial landscape and offered a forward‐looking perspective on how these innovations may drive OVT toward broader clinical applications. Future progress in OVT will depend on improving delivery methods, enhancing combination therapies, and implementing biomarker‐guided treatments.

## 2 OV‐Mediated Oncolysis and Antitumor Mechanisms

OVs exert antitumor effects through viral infection, selective replication in tumor cells, direct oncolysis, and the activation of antitumor immune responses. These effects are influenced by intrinsic viral properties as well as the molecular characteristics of tumor cells and the TME. This section introduces the major classes of OVs based on their genomic composition and structural features, followed by a description of the key steps in viral infection, including receptor recognition, cellular entry, and genome replication. The molecular basis of tumor selectivity and oncolytic activity is then discussed, with particular attention to defects in antiviral signaling pathways and oncogenic signaling that facilitate viral replication in tumor cells. Finally, the mechanisms by which OV infection stimulates innate and adaptive immune responses within the TME are summarized.

### 2.1 Classification of OVs

**FIGURE 1:**
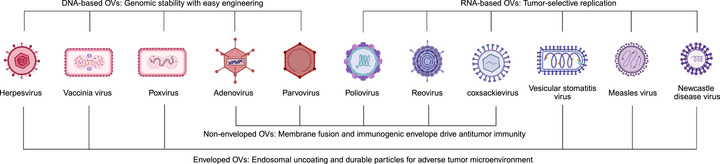
Classification of OVs by genome type and viral structure. OVs are grouped into DNA‐based and RNA‐based viruses and further subdivided into enveloped and nonenveloped categories. *Abbreviation*: OV, oncolytic virus.

OVs, which replicate in tumor cells and exert both direct oncolysis and immune modulation, have emerged as integral components in modern cancer immunotherapies [10]. Clinically relevant and translationally advanced OVs can be classified according to their genomic compositions as DNA‐ or RNA‐based viruses [11] and further distinguished as enveloped or nonenveloped based on their viral structure (Figure 1) [4]. DNA‐based OVs, including HSV, adenovirus, poxvirus, vaccinia virus (VV), and parvovirus, exhibit high genomic stability and substantial transgene capacity and are well‐established genetic engineering platforms [7]. These features enable tumor‐selective replication via promoter‐driven regulation or targeted gene deletions while ensuring viral stability and an acceptable safety profile [12]. In contrast, RNA‐based OVs, such as reovirus, measles virus (MV), Newcastle disease virus (NDV), vesicular stomatitis virus (VSV), and engineered poliovirus, generally replicate rapidly and display strong cytolytic activity [7]. Their replication is favored in tumor cells with impaired interferon (IFN) signaling, enabling selective amplification within malignant tissues [13, 14, 15].

From a structural perspective, enveloped viruses (e.g., HSV, VSV, NDV, MV, and poxviruses) typically enter host cells via envelope–plasma membrane fusion. Their lipid envelopes and surface glycoproteins strongly stimulate dendritic cells (DCs) and innate immune pathways, thereby enhancing systemic antitumor immune activation. In contrast, nonenveloped viruses (e.g., adenovirus, reovirus, and parvovirus) primarily depend on receptor‐mediated endocytosis, followed by protease‐dependent, stepwise uncoating within acidic endosomes or lysosomes. Their greater particle stability provides an adaptive advantage in the acidic, hypoxic, and protease‐rich TME (Figure 1). Therefore, the therapeutic efficacy and translational potential of OVs in cancer immunotherapy are fundamentally determined by their genomic architecture and structural morphology, which together mediate viral entry, replication kinetics, tumor selectivity, and immune activation within the TME.

### 2.2 Infection Mechanisms of OVs

OV infectivity is determined by virion architecture, genomic features, and antiviral signaling pathways. Structurally, OVs consist of a nucleocapsid enclosing a DNA or RNA genome, which may be linear or circular, single‐ or double‐stranded, and positive‐ or negative‐sense. Some viruses additionally acquire a host‐derived lipid envelope [4, 10, 16]. Genome‐encoded structural proteins form capsids, receptor‐binding domains, and envelope glycoproteins, whereas nonstructural proteins drive viral replication, modulate host signaling pathways, evade immune detection, and reprogram the cellular microenvironment. The virion structure plays a central role in viral stability, host tropism, and cell entry.

Nonenveloped OVs utilize rigid protein capsids that confer high physical stability under extracellular stress conditions [17, 18]. The geometric arrangement of capsid subunits shapes the spatial presentation of surface‐binding domains, thereby dictating host specificity and entry efficiency. This principle is exemplified by the adenovirus hexon, penton, and fiber knob proteins, which work together to mediate receptor recognition and initiate endocytosis [19, 20, 21]. In contrast, enveloped OVs are surrounded by a lipid membrane embedded with organized glycoproteins that facilitate receptor engagement, membrane fusion, immune modulation, and cell‐to‐cell spread, though this comes at the expense of increased sensitivity to temperature, pH, and environmental instability. Together, these structural features influence viral entry, replication, and tumor‐targeting potential [22, 23, 24].

**FIGURE 2:**
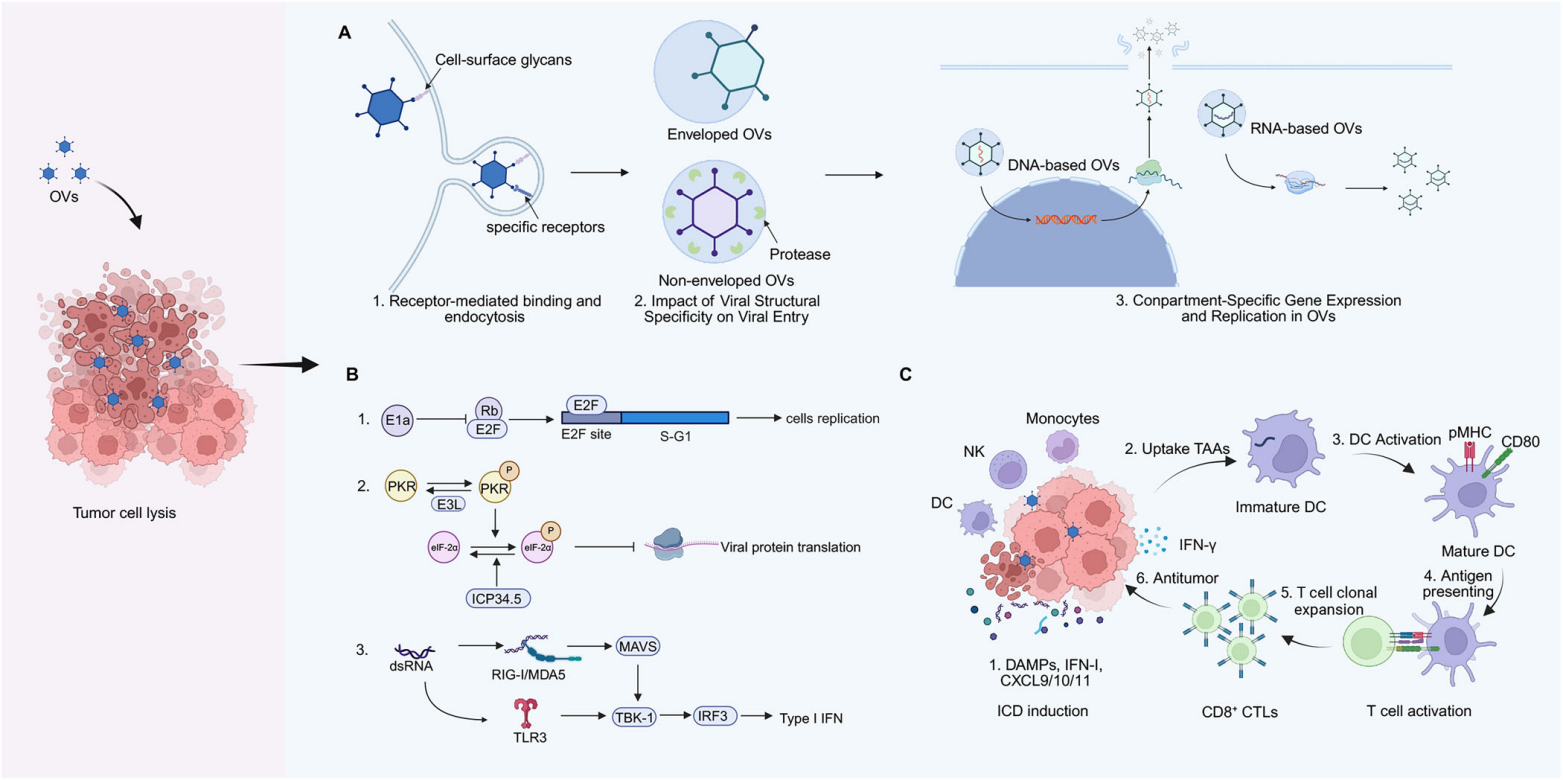
Oncolytic mechanisms of OVs in tumor cell lysis and antitumor effects. (A) OVs infection is initiated by low‐affinity interactions with cell‐surface glycans, followed by high‐affinity engagement of specific transmembrane receptors. Nonenveloped OVs enter tumor cells predominantly via receptor‐mediated endocytosis and undergo protease‐dependent uncoating within endosomal compartments. By contrast, enveloped OVs deliver nucleocapsids to the cytoplasm through membrane fusion at the plasma membrane or within endosomes after internalization. Genome type determines the site of viral replication, with DNA‐based OVs replicating in the nucleus and RNA‐based OVs replicating in the cytoplasm. (B) Aberrantly activated oncogenic signaling pathways in tumor cells, including the PI3K–AKT–mTOR and RAS–MAPK cascades as well as MYC signaling, support OV gene expression and viral protein translation, thereby enabling efficient replication in cancer cells. (C) OV‐induced antitumor immunity. OV infection triggers ICD and the release of PAMPs and DAMPs, TAAs and Type I interferons, thereby promoting immune cell activation within the TME (1). DCs uptake released antigens and undergo maturation (2,3), followed by migration to draining lymph nodes, where tumor antigens are presented to CD8^+^ T cells (4). Antigen presentation together with costimulatory signaling drives CD8^+^ T cell activation and clonal expansion (5). Activated CD8^+^ CTLs subsequently infiltrate tumor tissue and mediate antitumor cytotoxic responses (6). *Abbreviations*: OV, oncolytic virus; PI3K, phosphoinositide 3‐kinase; AKT, protein kinase B; mTOR, mechanistic target of rapamycin; RAS, rat sarcoma; MAPK, mitogen‐activated protein kinase; MYC, myelocytomatosis oncogene; ICD, immunogenic cell death; PAMP, pathogen‐associated molecular pattern; DAMP, damage‐associated molecular pattern; TAA, tumor‐associated antigen; TME, tumor microenvironment; DC, dendritic cell; CTL, cytotoxic T lymphocyte.

OV infection of tumor cells typically proceeds through a coordinated two‐step attachment process. In the initial phase, viral particles establish low‐affinity interactions with cell‐surface glycans, such as sialic acid or heparan sulfate proteoglycans, via capsid or glycoprotein components. This preliminary tethering concentrates the virions at the plasma membrane and promotes subsequent high‐affinity binding to specific transmembrane receptors (Figure 2A). Representative examples include adenovirus fiber knob binding to Coxsackievirus–adenovirus receptor or CD46, reovirus σ1 engagement of JAM‐A [25], MV hemagglutinin targeting SLAMF1 or nectin‐4 [26], and HSV glycoprotein D interacting with nectin‐1 or HVEM [27, 28]. Accordingly, high receptor expression on tumor cells provides a molecular basis for OV tropism and tumor selectivity.

Viral entry and uncoating are tightly regulated by virion structure (Figure 2A). Nonenveloped viruses primarily enter target cells via receptor‐mediated endocytosis through clathrin‐dependent or caveolae‐mediated pathways, followed by acidification‐dependent uncoating within endosomal compartments. The acidic endosomal environment activates proteases such as cathepsins B and L, which sequentially cleave capsid subunits and trigger conformational rearrangements that generate membrane‐penetrating subviral intermediates. This process is particularly critical for double‐stranded RNA (dsRNA) viruses such as reovirus [29, 30].

Enveloped viruses, by contrast, utilize membrane fusion to deliver their nucleocapsids into the cytoplasm. Depending on the virus‐specific entry pathways, fusion may occur directly at the plasma membrane upon receptor engagement or following endocytosis, where low pH‐induced conformational changes in envelope glycoproteins mediate fusion with the endosomal membranes. This mechanism is essential for enveloped OVs, such as HSV and VSV [31, 32, 33].

After uncoating, the viral genomes reach their replication sites. Most DNA‐based OVs replicate and transcribe their genomes in the host cell nucleus and exploit nuclear localization signals, nuclear pore complexes, and importin‐dependent transport pathways to access the nuclear compartment [34]. Poxviruses, however, are a notable exception, as they replicate exclusively in the cytoplasm [35]. In contrast, RNA‐based OVs typically complete the gene expression and replication processes within the cytoplasm, driven by virally encoded RNA‐dependent RNA polymerases.

Collectively, the OV infection process represents a highly coordinated, multistep biological event. This complexity is shaped by intrinsic viral properties such as genomic arrangement, replication strategies, capsid or envelope features, and progeny release mechanisms. Concurrently, molecular and immunological alterations within tumors create a permissive environment that promotes selective infectivity. These interrelated insights deepen our understanding of OV biology and provide a rational foundation for enhancing its clinical translation and therapeutic potential.

### 2.3 Oncolytic Mechanism and Tumor Selectivity of OVs

OVs exert their therapeutic effects primarily through selective replication within tumor cells and the subsequent induction of both local and systemic antitumor immune responses. This selective tropism and immunogenic capacity arise from multiple interrelated factors: inherent antiviral immune deficiencies in tumor cells, mutation‐driven dysregulation of signaling pathways, abnormal expression of viral receptors on malignant cell surfaces, and an immunosuppressive yet protease‐rich TME.

In normal cells, antiviral defense is triggered by pattern‐recognition receptors (PRRs), such as retinoic acid‐inducible gene I (RIG‐I), melanoma differentiation‐associated protein 5 (MDA5), toll‐like receptor 3 (TLR3), protein kinase R (PKR), and the cyclic GMP–AMP synthase–stimulator of IFN genes (cGAS–STING) pathway [36]. These sensors initiate signaling cascades that activate IFN regulatory factors 3 and 7 and nuclear factor kappa B to drive IFN production, which in turn induces IFN‐stimulated genes to suppress viral protein synthesis and limit viral replication [37, 38]. In contrast, tumor cells often exhibit widespread defects in these protective mechanisms, including PKR dysfunction, downregulation of the cGAS–STING pathway, reduced responsiveness to IFN receptors, silencing of the Janus kinase‐signal transducer and activator of transcription pathway, and promoter methylation of PRR [39, 40, 41]. These impairments compromise antiviral surveillance and create a permissive environment that supports substantially higher levels of OV replication in malignant cells than those in normal tissues. In addition, oncogenic signaling enhances viral permissiveness. Constitutive activation of pathways such as Ras‐mitogen‐activated protein kinase, phosphoinositide 3‐kinase–protein kinase B–mammalian target of rapamycin (PI3K–Akt–mTOR), or Myc not only drives uncontrolled proliferation but also establishes a metabolic state favorable for viral genome replication and protein synthesis [42, 43]. For instance, Ras activation counteracts PKR‐mediated translational suppression, enabling efficient amplification of RNA viruses such as reovirus and VSV [7]. Meanwhile, PI3K–Akt signaling enhances cell survival, and mTOR‐dependent metabolic reprogramming supplies the biosynthetic resources necessary to sustain viral replication [44].

The replication strategies of OVs during tumor lysis vary significantly across different classes, which in turn shape their behavioral profiles within the TME. DNA‐based viruses such as adenovirus, HSV, and VV typically exploit tumor‐associated cell cycle dysregulation for efficient replication by utilizing mechanisms such as Rb–E2F pathway defects [45], ICP34.5‐mediated PKR suppression [46], and E3L‐mediated dsRNA signaling blockade [47], respectively (Figure 2B). Their replication often integrates closely with the host DNA synthesis machinery in the nucleus or cytoplasm, conferring a natural tropism for actively proliferating malignant cells. However, RNA‐based viruses exhibit rapid cytoplasm‐focused replication independent of the cell cycle and rely on viral RNA polymerases for genome amplification. The generation of immunogenic dsRNA and viral ribonucleoprotein complexes robustly activates RIGI/MDA5 [48], TLR3 [49], and Type I IFN pathways (Figure 2B), promoting direct tumor lysis while concurrently enhancing DC maturation, antigen cross‐presentation, and T‐cell activation [50]. This dual effect enables RNA‐based viruses to reshape the immunologically silent TME into an inflammatory state that is capable of eliciting systemic antitumor immunity. Furthermore, structural distinctions, such as the envelope glycoproteins of enveloped viruses that mediate membrane fusion and syncytia formation [51, 52] and the protease‐dependent uncoating of nonenveloped viruses facilitated by tumor‐abundant proteases [53], further refine viral tropism, stability, and spread within the tumor stroma.

### 2.4 Antitumor Immunity Triggered by OVs

The capacity of OVs to convert immunologically quiescent (“cold”) tumors into inflamed (“hot”) lesions is mechanistically rooted in their ability to induce ICD (Figure 2C). Viral replication within tumor cells triggers the lytic release of damage‐associated molecular patterns (DAMPs) such as ATP, HMGB, and exposed calreticulin [54, 55]. These DAMPs synergize with virus‐derived pathogen‐associated molecular patterns (PAMPs) to enhance the maturation and activation of antigen‐presenting cells, notably DCs. TAAs and neoantigens released during this process are subsequently taken up, processed, and presented to T cells via the major histocompatibility complex (MHC)‐I and MHC‐II pathways. This enhanced antigen presentation initiates the clonal expansion and effector differentiation of CD8^+^ cytotoxic T cells, thereby establishing a tumor‐specific adaptive immune response [56].

In parallel, the virus‐elicited IFN‐I response, along with the upregulated chemokines, such as CXCL9 and CXCL10, drives the substantial recruitment of effector T cells and natural killer (NK) cells to the tumor site [57, 58, 59]. This immune cell influx actively remodels the local microenvironment while enhancing tumor immunogenicity through IFN‐I‐mediated upregulation of MHC‐I expression in tumor cells, further promoting their recognition and elimination by cytotoxic T cells. In addition, OVs attenuate immunosuppressive circuits within the TME by suppressing myeloid‐derived suppressor cells (MDSCs) [60] reducing regulatory T cell (Treg) activity [61]. As effector T cells expand and establish an immunological memory, the antitumor response extends to noninfected tumor foci, resulting in a potent “bystander killing” effect that significantly enhances the overall therapeutic outcome.

Overall, OVs initiate a progressive, multistage immune cascade, driven by viral replication, which is amplified through innate immunity and culminates in adaptive responses. This dual capability to lyse tumor cells directly while simultaneously modulating immune responses defines their unique therapeutic profile and provides a mechanistic basis for their strong synergy with ICIs and other immunotherapeutic strategies. Collectively, these properties underscore the capacity of OVs to orchestrate coordinated innate and adaptive immune responses in the TME.

## 3 Restrictive Factors for Virotherapy

Although OVs possess distinct advantages in reshaping the immune microenvironment and elicit immune‐mediated tumor control, they still face substantial barriers to clinical efficacy owing to host antiviral immunity, solid tumor characteristics, and immunosuppressive microenvironments. Rather than acting in isolation, these constraints operate at multiple levels, from systemic delivery and intratumoral spread to antiviral and immunosuppressive circuits within the TME, thereby limiting effective tumor exposure, viral amplification, and downstream antitumor immunity.

**FIGURE 3:**
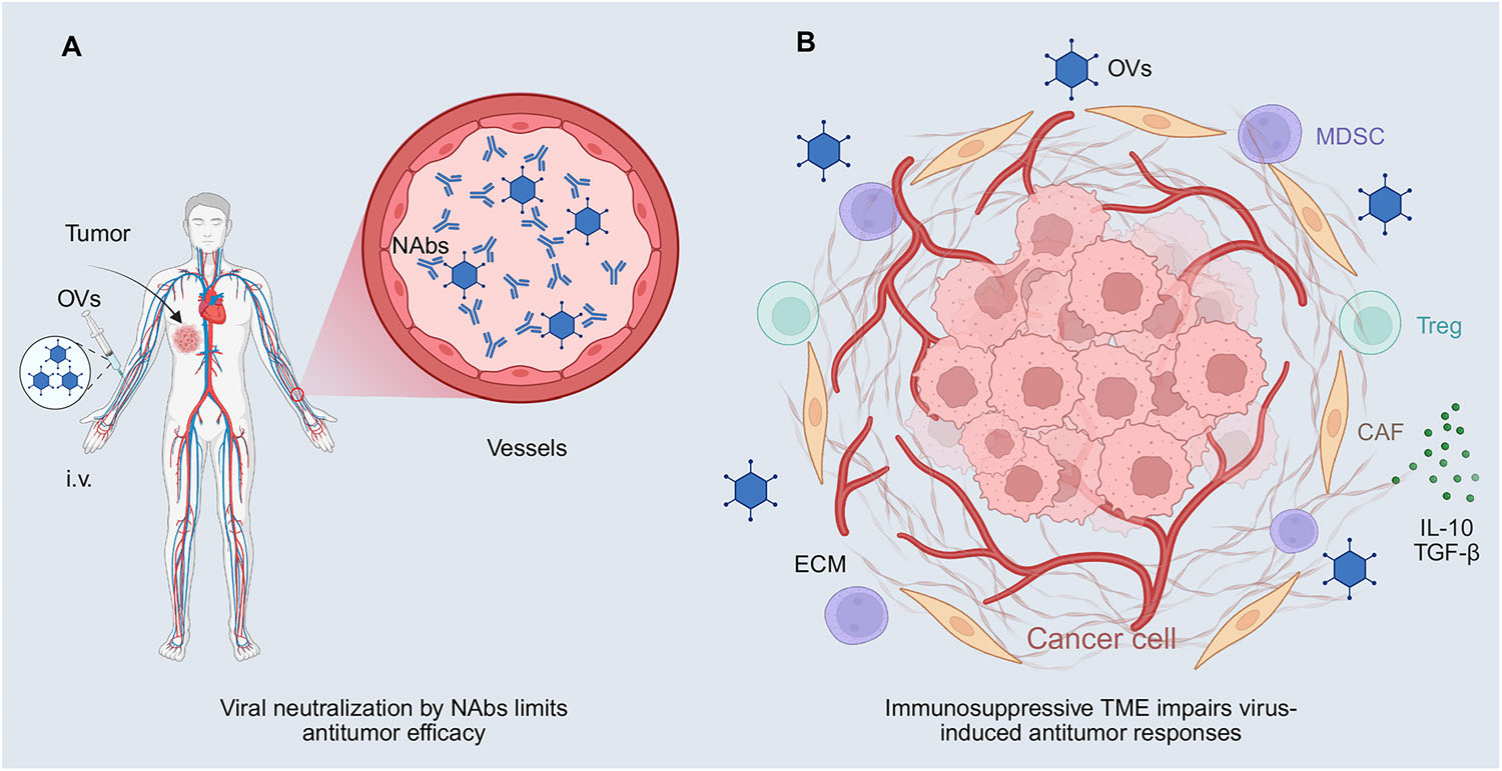
Immunological clearance and tumor microenvironmental suppression: key challenges limiting the clinical application of OVs. (A) Systemically administered OVs via i.v. injection are rapidly neutralized by pre‐existing NAbs, reducing therapeutic efficacy. Innate immune responses further accelerate viral clearance, limiting intravascular viral availability. (B) Barriers to OVs efficacy within the TME. Following tumor entry, OVs is confronted with both structural and immunological constraints. Aberrant tumor vasculature and a dense ECM limit viral extravasation, intratumoral penetration, and uniform distribution. Concurrently, an immunosuppressive TME enriched in Tregs, MDSCs, CAFs, and inhibitory cytokines, including IL‐10 and TGF‐β, impairs DCs maturation, antigen presentation, and effector T cell activity. Collectively, these factors attenuate OVs‐induced antitumor immunity and constrain therapeutic efficacy, particularly in immune‐evasive or poorly accessible tumors. *Abbreviations*: OV, oncolytic virus; i.v., intravenous; NAb, neutralizing antibody; TME, tumor microenvironment; ECM, extracellular matrix; Treg, regulatory T cell; MDSC, myeloid‐derived suppressor cell; CAF, cancer‐associated fibroblast; IL‐10, interleukin‐10; TGF‐β, transforming growth factor‐beta; DC, dendritic cell.

A primary challenge lies in pre‐existing immune responses, which critically hamper systemic delivery (Figure 3A). Many naturally occurring OVs exhibit high exposure rates in the general population, leading to the widespread presence of neutralizing antibodies even before treatment initiation. Circulating immunoglobulin (Ig) G and IgA antibodies rapidly clear viral particles within minutes, shortening circulation time and limiting tissue access. Even when viruses evade antibody neutralization, they are subjected to complement‐mediated opsonization and clearance by the mononuclear phagocytic system, thereby reducing the effective dose delivered to the tumor [62, 63]. This robust host immune landscape not only diminishes the efficacy of single‐dose administration but also limits the feasibility of repeated dosing strategies, contributing to variable and often limited systemic responses.

The anatomical and biophysical characteristics of solid tumors create structural barriers to viral distribution within the tumors (Figure 3B). Abnormal and highly tortuous vascular networks impede effective viral extravasation, whereas a dense extracellular matrix (ECM), elevated interstitial pressure, and poorly perfused regions collectively hinder the uniform spread of the virus throughout the tumor tissue. Consequently, viral propagation tends to be uneven and is often restricted to peripheral or accessible areas, with deeper, fibrotic, or hypoxic zones remaining poorly infected. This physical containment represents a key mechanism underlying the inconsistent therapeutic outcomes in solid tumors.

Furthermore, the immunosuppressive TME weakens virus‐induced immune activation (Figure 3B). Enrichment of Tregs, MDSCs, and M2 macrophages, along with sustained expression of inhibitory cytokines such as interleukin (IL)‐10 and transforming growth factor (TGF)‐β, suppresses inflammatory signaling and antigen presentation triggered by OVs. Although viral infections activate PRRs and initiate IFN‐I responses, these signals often fail to evolve into robust effector T cell infiltration or systemic antitumor immunity within an immunosuppressive milieu. In certain tumor subtypes, dominant immunosuppressive networks may even counteract the immunogenic effects of viral therapy, resulting in localized cytolysis without broader immune reprogramming.

Collectively, these multilayered barriers represent major biological constraints for OVT. In response, current research is increasingly focusing on innovative delivery and combinatorial strategies. These strategies include the use of cellular carriers and nanoparticle delivery systems to enhance tumor‐targeted delivery and evade immune clearance, as well as combining OVs with ICIs, metabolic modulation, or chemoradiotherapy to overcome suppressive barriers in the TME. Through these advances, OVT is moving beyond a standalone modality toward integrative strategies that combine cellular and nanoparticle‐based delivery with rational combinatorial immunotherapeutic approaches. This transformation aims to reshape the complex interactions with host immunity and the TME, thereby achieving more durable and comprehensive antitumor effects.

## 4 Optimization of Delivery Systems

As research on OVs progresses, optimizing delivery methods has become crucial for enhancing in vivo performance and clinical potential. Various supportive delivery strategies have been explored to overcome the limitations of conventional administration and improve therapeutic outcomes. These strategies can be classified into three primary categories: cell‐based delivery, nanomaterial‐assisted carriers, and extracellular vesicle (EV)‐mediated delivery. Each approach leverages distinct biological or material properties to enhance viral stability, optimize distribution profiles, and prolong therapeutic persistence. However, all approaches face inherent limitations that should be carefully considered to maximize therapeutic benefits and minimize risks (Table 1).

**TABLE 1:** Comparative analysis of delivery strategies for OV.

Delivery strategy	Advantages	Limitations	References
NSCs	Intrinsic tumor tropism toward brain tumors; Ability to cross the BBB; Facilitation of localized OV release and enhanced intratumoral viral activity	Primarily applicability to CNS tumors; Challenges in immune compatibility and large‐scale manufacturing	[64, 65, 66]
MSCs	Ease of isolation and systemic delivery; Strong inherent tumor‐homing capacity; Inherently low immunogenicity; Potent antitumor immune activation	Context‐dependent dual roles in tumor biology; Potential oncogenic risks	[67, 68, 69, 70, 71, 72, 73, 74]
CAR‐T cells	Antigen‐specific tumor targeting independent of MHC; Enhanced antigen release and immune infiltration; Generation of dual‐specific T cells recognizing tumor and viral antigens	On‐target/off‐tumor toxicity; Limited persistence and T‐cell exhaustion	[75, 76, 77, 78, 79, 80, 81, 82, 83, 84, 85, 86, 87, 88]
NK cells	Innate cytotoxicity and cytokine secretion; OV enhanced tumor susceptibility to NK killing	Functional suppression within immunosuppressive TME; Inefficient expansion at clinical scale; Need to balance NK cell activation and NK cell expansion	[89, 90, 91, 92, 93]
EVs	High biocompatibility and low immunogenicity; Effective viral shielding and prolonged circulation	Insufficient intrinsic tumor targeting; Rapid clearance by the mononuclear phagocyte system; Lack of standardized production and quality control	[94, 95, 96, 97, 98, 99, 100]
Liposome	Biocompatible with controllable release; Protective against neutralizing antibodies; Enhanced in circulation and tumor accumulation via PEGylation and ligand modification	Immune‐mediated clearance and structural instability; Limited tumor penetration; Need further optimization for targeting and viral stability	[101, 102, 103, 104, 105, 106, 107, 108, 109]
Polymer	Tunable physicochemical properties; Stimulus‐responsive release; Ligand‐guided targeting for enhanced specificity; minimized off‐target transduction	Complex structure may hamper translational characterization; Their therapeutic profiles present challenges to efficacy, scalability, and safety.	[110, 111, 112, 113, 114, 115, 116, 117]

### 4.1 Cell‐Based Delivery Strategies

Cell‐based delivery strategies have attracted increasing attention owing to their unique biological advantages, and several studies have explored their potential applications [75, 118, 119]. As natural carriers, these cells shield viruses during systemic transit, maintaining their structural integrity and infectivity. Certain cell types can also create a supportive microenvironment that further facilitates viral delivery and localized amplification, thereby enhancing the overall delivery efficiency and potency.

Effective cell‐based carriers typically share several essential attributes, including the ability to internalize and stably encapsulate viruses while limiting immune exposure, inherent tumor tropism, directional migration for targeted delivery, and immunomodulatory or evasive properties that sustain viral persistence in vivo. Cell types such as stem and immune cells naturally exhibit tumor‐homing properties, allowing them to actively migrate to tumor sites in response to microenvironmental signals. These cells can also penetrate physical barriers within tumors, promoting deep tissue penetration of the virus and significantly boosting local infection rates and viral concentration. Additionally, cell‐mediated delivery helps confine viral distribution to target tissues, reducing off‐target exposure in healthy organs and improving therapeutic selectivity and safety. Certain carrier cells also possess intrinsic antitumor or immunoregulatory functions. When combined with OVs, these properties can produce synergistic effects that amplify antitumor immunity and improve the overall treatment efficacy.

**FIGURE 4:**
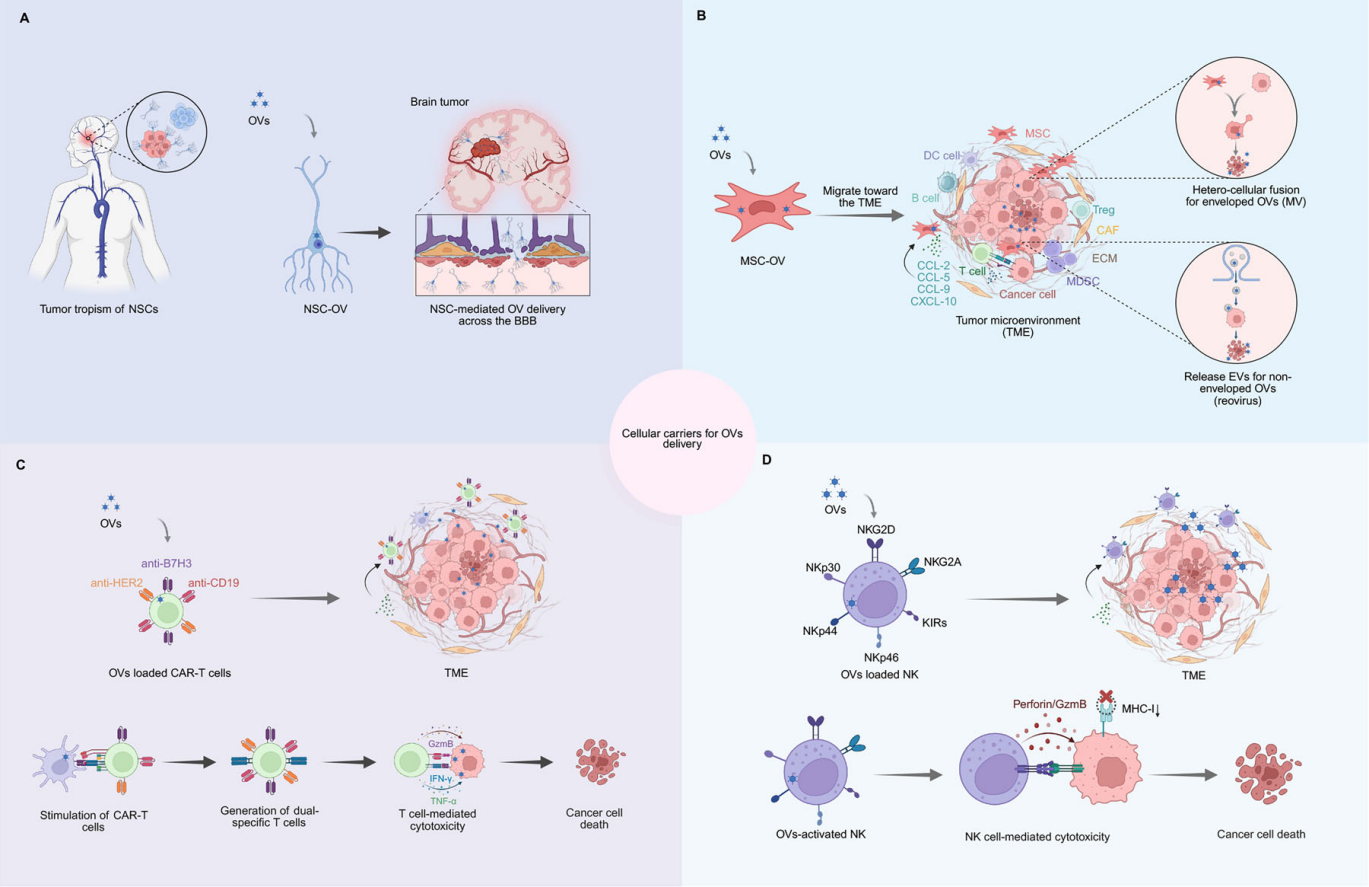
Cell‐based delivery platforms for OVs cancer therapy. (A) NSCs cross the BBB to deliver OVs to CNS tumors. Their tumor‐tropic migration enables localized viral replication and immune activation within glioblastomas while minimizing systemic exposure. (B) MSCs exhibit potent tumor‐homing capacity through CCL‐2/5/9, CXCL‐10. Their delivery mechanism depends on viral structure: enveloped viruses (e.g., measles virus) deliver via hetero‐cellular fusion, while nonenveloped viruses (e.g., reovirus) are secreted in EVs. (C) Antigen‐specific CAR‐T cells targeting HER2, B7‐H3, or CD19 serve as dual‐function carriers that deliver viruses while maintaining tumor‐targeting and cytotoxic capabilities. Following tumor infiltration, virus release triggers ICD, liberating tumor antigens and activating endogenous T‐cell responses to generate bispecific T cells. This approach employs virus as “Trojan horses” to overcome immune evasion and enhance antitumor immunity through combined CAR‐T cell targeting and viral‐mediated immune activation. (D) NK cells effectively deliver OVs through innate tumor‐recognition mediated by activating receptors (NKG2D, NKp30/44/46) and inhibitory receptors (NKG2A, KIRs). In the TME, OVs triggers ICD that upregulates NK‐activating ligands on tumor cells while simultaneously enhancing NK cytotoxicity via increased perforin/granzyme B release. *Abbreviations*: OV, oncolytic virus; NSC, neural stem cell; BBB, blood–brain barrier; CNS, central nervous system; MSC, mesenchymal stem cell; EV, extracellular vesicle; CAR‐T, chimeric antigen receptor T cell; ICD, immunogenic cell death; TME, tumor microenvironment; NK, natural killer; NKG2D, natural killer group 2 member D; NKp, natural killer cell p receptor; NKG2A, natural killer group 2 member A; KIR, killer cell immunoglobulin‐like receptor.

This section reviews recent advances in cell‐based delivery strategies (Figure 4), highlights their clinical promise, and discusses the theoretical and practical foundations for the broader implementation of OVs in cancer therapy.

#### 4.1.1 Neural Stem Cells as Cellular Carrier for OV Delivery

Neural stem cells (NSCs) hold considerable promise as cellular carriers for OVs, largely because of their intrinsic tumor‐tropic properties. Their unique capacity to cross the blood–brain barrier (BBB) makes them particularly valuable for targeting aggressive brain malignancies, such as glioblastoma multiforme [64]. Upon reaching the tumor site, NSCs facilitate the localized release of OVs into the TME, where the viruses replicate, induce direct oncolysis, and stimulate antitumor immune responses (Figure 4A).

NSCs have been used to deliver modified adenoviruses, such as those incorporating a fiber modified with seven lysine residues (NSC‐CRAd‐S‐pk7), designed to enhance viral entry into tumor cells [65]. Clinical studies indicate that this approach is generally well tolerated and may provide therapeutic benefit in patients with advanced malignant gliomas [120]. NSC‐mediated delivery significantly enhances viral replication at the tumor site and stimulates antitumor immune responses, resulting in improved treatment outcomes. The inherent capacity of NSCs to traverse the BBB addresses a major challenge in central nervous system drug delivery, enabling targeted accumulation of therapeutic viruses while minimizing off‐target exposure. Recently, the intracerebroventricular (ICV) administration of NSC‐delivered OVs has been explored to improve tumor accessibility and distribution throughout the brain. Using preclinical in vivo models, Chai et al. demonstrated that ICV‐delivered NSC‐OVs achieve broader tumor coverage and sustained intratumoral viral activity while simultaneously modulating the immune microenvironment, thereby supporting enhanced antitumor immune responses [119]. The replication of this virus relies on elevated thymidine kinase 1 expression in tumor cells. NSC‐mediated delivery concentrates viral activity at metastatic sites [66]. Similarly, NSCs have been used to effectively deliver myxoma viruses to ovarian cancer cells, supporting sustained viral replication and selective tumor lysis [66, 121]. Taken together, these results highlight the versatility of the NSC platform to deliver diverse OVs across various tumor types.

Mesenchymal stem cells (MSCs) have emerged as a promising stem cell platform for OV delivery, particularly in non‐neural malignancies. Compared with NSCs, MSCs offer advantages such as ease of isolation, systemic delivery potential, and broad tumor‐homing capability. Their ability to support viral replication has been confirmed in preclinical and clinical studies, extending the scope of virotherapy beyond the central nervous system. Taken together, NSCs and MSCs represent complementary strategies for targeted OV delivery across a range of tumor types, although their effectiveness may depend on the tumor context and several translational challenges, including potential immune rejection and difficulties in large‐scale manufacturing, must be considered. The following sections examine the application of MSCs in more detail.

#### 4.1.2 MSCs as Cellular Carriers for OV Delivery

MSCs are nonhematopoietic stem cells that can be isolated from various tissues, including the bone marrow, adipose tissue, dental pulp, placenta, amniotic fluid, umbilical cord, Wharton's jelly, and umbilical cord blood [67]. Despite variations in tissue origins and genetic backgrounds, cultured MSCs generally share common phenotypic and functional characteristics. They typically express surface markers, such as CD105, CD73, and CD90, but lack human leukocyte antigen Class II (HLA Class II) antigens and costimulatory molecules, including CD40, CD80, and CD86 [122]. This immunophenotype confers low immunogenicity and supports their use in both autologous and allogeneic transplantation, with a reduced need for immunosuppression [68, 69]. In addition, MSCs exhibit multipotent differentiation potential toward osteoblasts, adipocytes, and chondrocytes under appropriate culture conditions. They also display tumor tropism, migrating toward tumor sites via chemokine signaling pathways, such as chemokine (C–C motif) ligand 2 (CCL‐2) [70], CCL‐5 [71], CCL‐9 [122], and CXCL10 [72], and interacting with components of the tumor ECM.

Notably, the role of MSCs in tumor progression is context dependent and often dualistic. Some studies have indicated that MSCs may support tumor growth by suppressing immune responses, promoting epithelial–mesenchymal transition, inhibiting tumor cell apoptosis, and stimulating angiogenesis and metastasis. These protumorigenic activities are thought to be influenced by the TME, which can reprogram MSCs to adopt tumor‐supportive phenotypes. Conversely, other evidence suggests that MSCs can exert antitumor effects, such as inhibiting angiogenesis, inducing cell cycle arrest, enhancing immune cell infiltration, and downregulating proliferation‐related pathways like PI3K/AKT and Wnt/β‐catenin [73, 74]. Despite this ongoing debate, preclinical and clinical studies support the use of MSCs as effective carriers of OVs (Figure 4B). Du et al. [123] demonstrated that HSV‐loaded MSCs effectively migrated to tumor sites and significantly prolonged survival in immunodeficient and immunocompetent melanoma metastasis models. When combined with an anti‐programmed death‐ligand 1 (PD‐L1) checkpoint inhibitor in immunocompetent mice, this approach further improved outcomes by increasing CD8^+^ T cell infiltration, enhancing IFN‐γ production, and extending survival. Similarly, MSC‐delivered adenoviruses have been shown to promote antitumor immunity and intratumoral leukocyte infiltration in experimental models. A Phase I clinical trial (NCT01844661) evaluating MSCs carrying the adenovirus Celyvir reported favorable safety, with no grade 2–5 adverse events observed [124]. Mechanistically, OV infection of MSCs can activate NF‐κB signaling, leading to the secretion of proinflammatory cytokines such as IL‐6, CXCL2, CXCL10, and CCL5 [125, 126]. These cytokines promote the infiltration of NK cells and T lymphocytes into the TME, indicating systemic immune activation.

MSCs facilitate OV delivery through hetero‐cellular fusion, particularly for enveloped viruses, and preclinical studies suggest that this can enhance systemic immune activation; however, most evidence is preclinical, and systemic immune responses in humans may differ (Figure 4B). Ong et al. [127] used MSCs to deliver MV and cocultured them with hepatocellular carcinoma cells in vitro. MSC‐mediated delivery enhanced syncytial formation, an effect not observed in nonenveloped viruses. Notably, MSC‐based delivery remained effective even in the presence of high titers of antimeasles antibodies. In patient‐derived hepatocellular carcinoma models, MSCs successfully targeted and delivered MVs to tumor lesions. These results are consistent with those of Castleton et al. [128], who demonstrated that MSC‐mediated delivery of the MV in a model of acute lymphoblastic leukemia significantly improved survival and enhanced antitumor efficacy compared with that of direct naked virus administration. Additionally, MSCs have been shown to effectively deliver nonenveloped reoviruses to tumors [128, 129]. A key mechanism involves the release of EVs that encapsulate the virus and facilitate its transfer to the target cells (Figure 4B) [94, 130]. This EV‐mediated route may shield the virus from immune clearance and enhance its accumulation and infectivity at the tumor site.

Genetic engineering strategies have been used to enhance the efficiency and safety of MSC‐based OV delivery. Yoon et al. [131] demonstrated that modifying the fiber domain of an adenovirus improved the infectivity of MSCs, enabling efficient viral replication within carrier cells. These virus‐loaded MSCs exhibited tumor tropism, leading to significant inhibition of hepatocellular carcinoma growth in vivo. In another study, Kaczorowski et al. [132] engineered an adenovirus by deleting the antiapoptotic gene E1B19K and inserting the TRAIL gene. MSCs carrying the modified virus were directed to pancreatic ductal adenocarcinoma stem cell‐derived xenografts, leading to reduced tumor volume, decreased proliferation markers (Ki67 and CD24), and increased caspase‐3 activity. Genetic modifications also enhanced viral release from MSCs without compromising their migratory capacity [133], highlighting a promising approach for augmenting the oncolytic potency of MSC‐delivered viruses.

Overall, MSC‐based delivery can concentrate therapeutic viruses at tumor sites and enhance the efficacy of OVT. However, further studies are warranted to improve MSC migration, adhesion, survival, and the prevention of premature senescence. The dual role of MSCs in tumor progression warrants further investigation to clarify the balance between their pro‐ and antitumorigenic effects. Future studies should explore genetic and pharmacological strategies to optimize MSC behavior and cytokine secretion profiles to maximize therapeutic benefits while mitigating potential oncogenic risks.

#### 4.1.3 Chimeric Antigen Receptor**‐T Cells as Cellular Carriers for OV Delivery**

Chimeric antigen receptor (CAR) T cells are genetically engineered to express synthetic receptors that specifically recognize tumor surface antigens, enabling the targeted elimination of target cells independent of MHC presentation [76]. Structurally, CARs consist of four key components, including an antigen‐binding domain, which is usually derived from a single‐chain variable fragment (scFv), a hinge region, a transmembrane domain, and an intracellular signaling domain that contains costimulatory domains, such as CD28 or 4‐1BB, along with a signaling domain, such as CD3ζ [77]. The scFv is generated by linking the variable regions of the heavy and light chains of the antibody, conferring specificity to TAAs. CAR‐T therapy has achieved notable success in hematologic malignancies such as leukemia and lymphoma [78, 79, 80]. However, the application of this therapy to solid tumors is still limited because of a series of complex challenges. Antigen escape is a major obstacle wherein tumor cells downregulate or alter the expression of target antigens, thereby evading CAR‐T cell recognition [81]. Another critical barrier arises even when CAR‐T cells reach the tumor site because their function can be severely impaired by the immunosuppressive TME, which is enriched with Tregs, MDSCs, tumor‐associated macrophages, and immunosuppressive cytokines [82, 83, 84, 85]. Combining CAR‐T therapy with OVs has emerged as a promising strategy to overcome these limitations. CAR‐T cells exhibit inherent tumor tropism, which makes them effective carriers for OV delivery (Figure 4C). OVs selectively lyse tumor cells at the tumor site, releasing TAAs and enhancing antigen presentation. This process stimulates a robust antitumor immune response and promotes lymphocyte infiltration, potentially converting immunologically “cold” tumors into “hot” immune‐responsive ones.

By leveraging the tumor‐targeting ability of CAR‐T cells, OVs can precisely accumulate within tumor tissues, integrating the antigen‐specific cytotoxicity of CAR‐T cells with the immunostimulatory effects of OVs to produce a durable and comprehensive antitumor response. Recent preclinical studies have shown that loading CAR‐T cells with VSV or reovirus preserves CAR surface expression and effector function and, in some settings, augments CAR signaling and cytotoxicity [63, 75, 86]. In another study, anti‐B7‐H3 CAR‐T cells carrying HSV displayed significantly improved antitumor activity without impairing T cell function [75].

CAR‐T cells have been used as carriers to deliver chemokine‐encoding OVs and enhance immune cell infiltration. An adenovirus encoding CXCL11 was shown to promote anti‐B7H3 CAR‐T cell migration into glioma tissues while reshaping the TME by increasing CD8^+^ T cells, NK cells, and M1 macrophages and reducing immunosuppressive Tregs and MDSCs [87]. Alternatively, combining MSC‐mediated OV delivery with CAR‐T cell therapy offers another promising strategy. MSCs were engineered to deliver adenovirus encoding IL‐12 and a PD‐L1 inhibitor gene. This combination significantly enhanced CAR‐T cell activation within the tumor and improved the local immune landscape when coadministered with anti‐human epidermal growth factor receptor 2 (HER2) CAR‐T cells in a lung cancer model [88]. Notably, the combination of CAR‐T cells with OVs, such as VSV and reovirus, has been shown to stimulate CAR‐T cells to express T cell receptors (TCRs) specific for virus‐associated antigens. This resulted in the generation of dual‐specific T cells that targeted both viral antigens and CD19 (Figure 4C) [76]. The experimental results demonstrated that this approach induced in vivo proliferation and a persistent memory phenotype of CAR‐T cells, significantly extending their survival in mouse models of melanoma and glioma. It also facilitated the in vitro expansion of anti‐CD19 CAR‐T cells with enhanced TCR reactivity against viral antigens and increased cytokine production. Therefore, for tumor cells that downregulate antigen expression, OVs may act as “Trojan horse antigens,” reintroducing immune targets to overcome antigen escape and restore antitumor immunity [134]. These results are predominantly preclinical, and clinical translation faces challenges such as antigen escape, CAR‐T exhaustion, and safety risks, including cytokine release and off‐tumor toxicity.

Despite these promising advances, several challenges remain. Identifying optimal antigen targets remains critical because they must enable precise tumor recognition while avoiding damage to healthy tissues. Although CD19 is a widely targeted antigen, its engagement risks B‐cell depletion and neurotoxicity. Ideally, target antigens should be highly tumor specific to minimize the risk of on‐target, off‐tumor toxicity. Furthermore, sustaining CAR‐T cell persistence and counteracting T cell exhaustion are essential for durable therapeutic efficacy [135, 136]. Developing strategies that extend CAR‐T cell survival and preserve their functional activity will be instrumental in realizing the full potential of combined CAR‐T cell and OV therapies.

#### 4.1.4 NK Cells as Cellular Carriers for OV Delivery

NK cells are innate immune effectors capable of eliminating malignant cells without prior antigen presentation. Derived from bone marrow hematopoietic stem cells, they circulate widely and exert cytotoxic functions through perforin–granzyme release while secreting immunomodulatory cytokines such as IFN‐γ and TNF‐α [89]. NK cell activity is tightly regulated by a balance between activating receptors, such as NK group 2 member D (NKG2D), and natural cytotoxicity receptors (NKp30, NKp44, and NKp46) and inhibitory receptors, including killer‐cell Ig‐like receptors and NK group 2 member A (NKG2A). These inhibitory receptors recognize MHC molecules in normal cells and prevent unintended damage [137, 138, 139]. Tumor cells often downregulate MHC expression to evade T cell surveillance, a strategy that paradoxically makes them more susceptible to NK cell‐mediated killing [140]. However, within the TME, NK cell function is frequently suppressed. Immunosuppressive mediators, such as TGF‐β, IL‐10, prostaglandin E2, and indoleamine 2,3‐dioxygenase, impair NK cell activity by reducing activating receptor expression, inducing functional dysfunction or hyporesponsiveness [141, 142, 143, 144]. In parallel, tumors exploit inhibitory pathways, such as the NKG2A–HLA‐E axis, to further impair NK cell cytotoxicity [90, 91, 145]. Therefore, reactivation of NK cells within the TME is essential for enhancing cancer immunotherapy.

OVs induce ICD in tumor cells, stimulating antitumor immune responses through multiple pathways. In particular, reoviruses increase tumor susceptibility to NK cell‐mediated killing by upregulating activating ligands such as NKG2D and downregulating MHC‐I expression [92]. This dual effect enhances NK cell recognition and elimination of tumor cells, thereby amplifying the overall antitumor immunity (Figure 4D). Therefore, OVs act as direct cytotoxic agents and can enhance NK cell‐mediated antitumor activity in preclinical models, though clinical efficacy remains to be established. To further optimize this therapeutic outcome, NK cell‐based delivery strategies have been developed, including NK cell‐mediated adenovirus delivery systems [146] and combination therapies using CAR–NK cells with HSV [147]. Importantly, emerging evidence indicates that coupling OVs with NK or CAR–NK cell platforms provides advantages beyond enhanced viral transport. For example, an IL‐15/IL‐15Rα‐armed OV (OV‐IL15C) combined with off‐the‐shelf epidermal growth factor receptor (EGFR)–CAR–NK cells demonstrated synergistic antitumor activity in glioblastoma, which was associated with increased intratumoral (intracranial) infiltration and activation of NK cells (and CD8^+^ T cells), as well as improved persistence of CAR–NK cells. Moreover, recent reviews of OV combination therapies have highlighted cellular immunotherapies, including CAR–NK cells, as rational partners capable of simultaneously improving delivery efficiency and amplifying antitumor immune responses [147].

Despite the promise of combined NK cell and OV‐based therapies, several challenges should be addressed before successful clinical translation. While IFN‐I signaling is essential for NK cell activation, it can also antagonize IL‐15‐driven NK cell expansion, highlighting the need to balance immune stimulation with impaired IL‐15‐dependent proliferation and altered NK cell responsiveness [93]. Moreover, the immunosuppressive TME continues to hinder NK cell efficacy through inhibitory pathways, such as NKG2A–HLA‐E, and inadequate chemokine‐mediated infiltration [148].

Both stem and immune cells exhibit pronounced tumor‐tropic properties and can serve as effective carriers for the targeted delivery of OVs to tumor sites. Compared with acellular delivery platforms, cell‐based systems can actively home to tumors and infiltrate heterogeneous TMEs, which may enhance intratumoral viral distribution in preclinical settings. Through their paracrine activities, stem cells can modulate antitumor immune responses; however, their context‐dependent immunoregulatory functions may also confer potential protumorigenic effects, making the balance between therapeutic efficacy and safety a critical and unresolved challenge. In contrast, immune cells represent a potentially promising delivery platform owing to their intrinsic tumoricidal activity and capacity to synergize with OV‐mediated immune activation. Nevertheless, immune cell‐based OV delivery remains largely experimental, with limited clinical evidence to date. From a translational perspective, both stem cell‐ and immune cell‐based strategies face substantial scalability and manufacturing challenges, including donor variability, ex vivo expansion, viral loading efficiency, and stringent quality control requirements. Given the substantial heterogeneity of solid tumors, future efforts should focus on expanding the repertoire of broadly applicable antigenic targets and exploring the use of CAR‐T and CAR–NK cells to deliver diverse classes of OVs, with the goal of further optimizing cell‐based viral delivery systems and advancing their clinical translation.

### 4.2 EV‐Based OV Delivery

Cell‐based carriers such as NSC, MSCs, CAR‐T cells, and NK cells have been widely used to deliver OVs with improved targeting and protection in vivo; however, they are not without limitations. Allogeneic cell products carry a risk of immune rejection and often require concurrent immunosuppression, which introduces additional safety and logistical complexities. Certain cell types, such as NK cells, are difficult to expand to clinically relevant numbers in vitro. Moreover, live‐cell therapies may trigger adverse effects ranging from cytokine release syndrome to potential tumor formation. To overcome these challenges, EVs have attracted increasing interest as alternative carriers for OV delivery. EVs are nanosized lipid bilayer particles secreted by nearly all cell types and carry diverse bioactive cargo, such as lipids, proteins, RNAs, and other metabolites, reflecting the composition of their parent cells [149]. Their inherent biocompatibility, low immunogenicity, and the ability to cross biological barriers make them attractive delivery platforms [95, 96]. Therapeutically, EVs can serve as targeted vehicles for antitumor agents or bioactive molecules, enhancing localized efficacy while minimizing off‐target effects [150, 151, 152].

**FIGURE 5:**
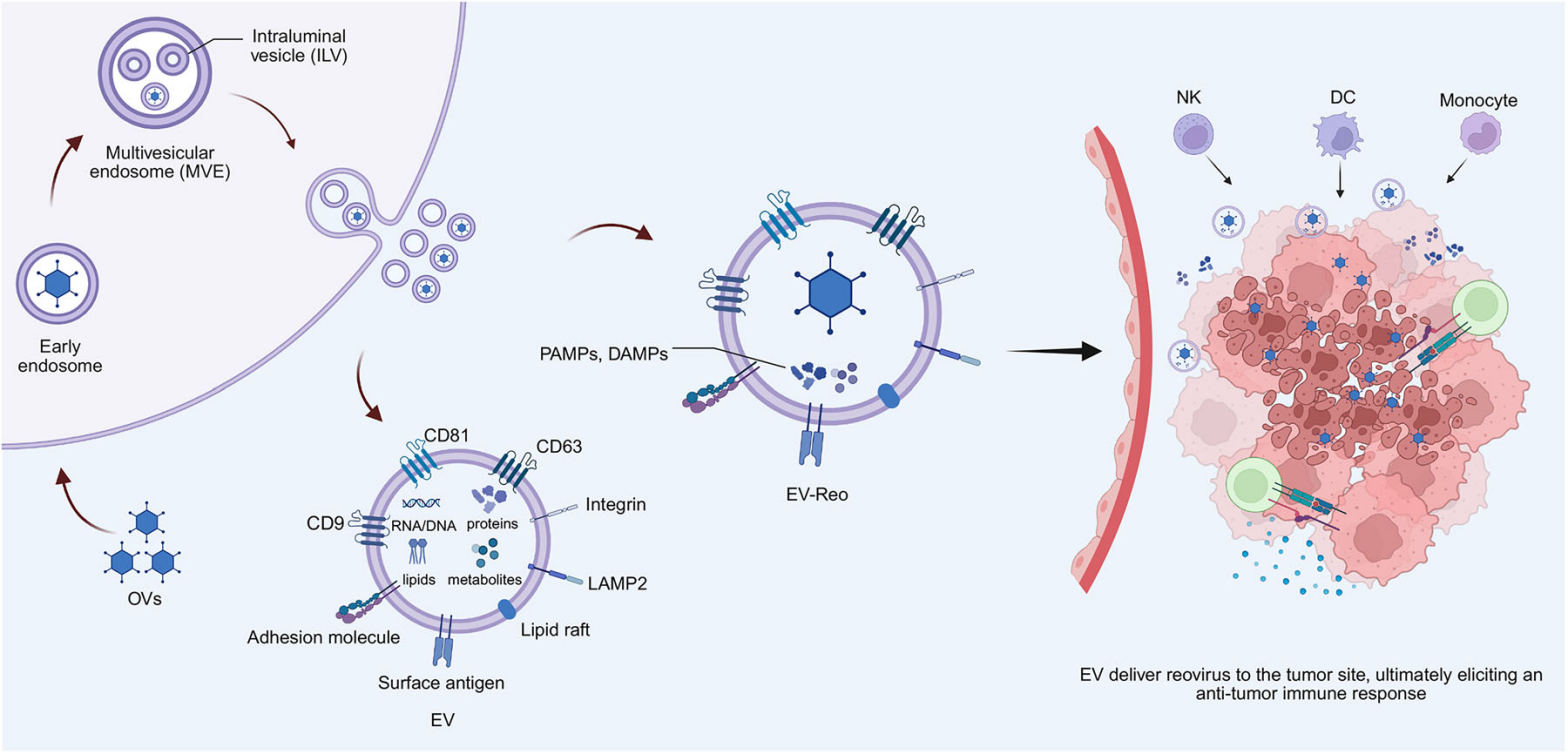
EV‐mediated OVs delivery and antitumor immune activation. EVs naturally encapsulate OVs during the endosomal maturation process, where viral particles in early endosomes become incorporated into ILVs of MVEs, ultimately being secreted as EV–OV complexes. These particles contain both intact virions and immunostimulatory PAMPs/DAMPs, and utilize inherent EV surface components, including tetraspanins (CD63, CD81, CD9), integrins, and adhesion molecules, for enhanced tumor targeting and biological barrier penetration. Following tumor cell uptake, the released OVs mediates direct oncolysis while concurrently activating NK cells, DCs and monocytes to establish sustained antitumor immunity. *Abbreviations*: EV, extracellular vesicle; OV, oncolytic virus; ILV, intraluminal vesicle; MVE, multivesicular endosome; PAMP, pathogen‐associated molecular pattern; DAMP, damage‐associated molecular pattern; NK, natural killer; DC, dendritic cell.

A recent study has highlighted EVs as promising mediators of OV‐based virotherapy (Figure 5) [153]. By encapsulating or associating with viral particles, EVs can shield the virus, prolong circulation time, enhance tumor targeting specificity, and activate antitumor immunity, as shown in preclinical models; clinical validation is pending [154, 155]. Surface molecules, such as CD63, CD9, CD81, LAMP2, integrins, cell adhesion proteins, and lipids, facilitate uptake by recipient cells [156, 157]. Notably, EVs derived from OV‐infected cells are slightly larger, likely because of viral encapsulation, but remain within the typical vesicle size range [158]. More broadly, EVs released from OV‐infected tumor cells can carry not only viral particles but also immunologically active cargo, such as PAMPs, DAMPs, and IFN‐I [154, 155]. This dual cargo supports enhanced tumor cell killing, immune activation, and sustained viral spread compared with that of the naked virus. Similarly, MSC‐derived EVs have been explored as OV carriers and may improve viral stability, prolong systemic persistence, and enhance delivery, even in tumors with low inherent viral susceptibility [94].

The source of the EVs critically influences their therapeutic profile. Tumor‐derived EVs often exhibit strong tumor‐targeting capabilities due to their origin but may present with higher immunogenicity, raising safety concerns. In contrast, MSC‐derived EVs display lower immunogenicity and are generally considered safer for clinical use, although they may have weaker intrinsic targeting abilities. Therefore, balancing targeting efficiency with safety is essential for effective OV delivery. Moreover, the systemic bioavailability of EV‐based delivery platforms remains limited because they are rapidly sequestered and cleared by the mononuclear phagocyte system. Rational engineering strategies, such as the incorporation of “don't eat me” signals including CD47, may mitigate immune recognition and phagocytic clearance, thereby extending circulatory persistence and improving in vivo delivery efficiency [97, 98]. Additionally, the intrinsic tumor‐targeting capacity of EVs is often insufficient, which necessitates surface engineering strategies, such as the conjugation of targeting ligands or antibodies against tumor‐specific receptors, including EGFR or HER2 [99, 100]. Accurately quantifying the targeting specificity and delivery efficiency of engineered EVs remains a major technical hurdle, limiting their optimization and clinical translation. Therefore, future studies should focus on standardizing EV production and quality control, improving targeting precision, and exploring synergistic combinations with other therapeutic modalities.

### 4.3 Nanoparticle‐Based OV Delivery

Nanoparticles have emerged as a promising platform for OV delivery [159, 160] because of their unique physicochemical properties. Their nanoscale dimensions, high surface area, and capacity for functionalization enable the efficient loading and targeted delivery of viral therapeutic agents and biomolecules [161, 162]. These characteristics facilitate improved tissue penetration, prolonged circulation, and controlled release kinetics at the target site, thus substantially enhancing the therapeutic efficacy while minimizing adverse effects.

**FIGURE 6:**
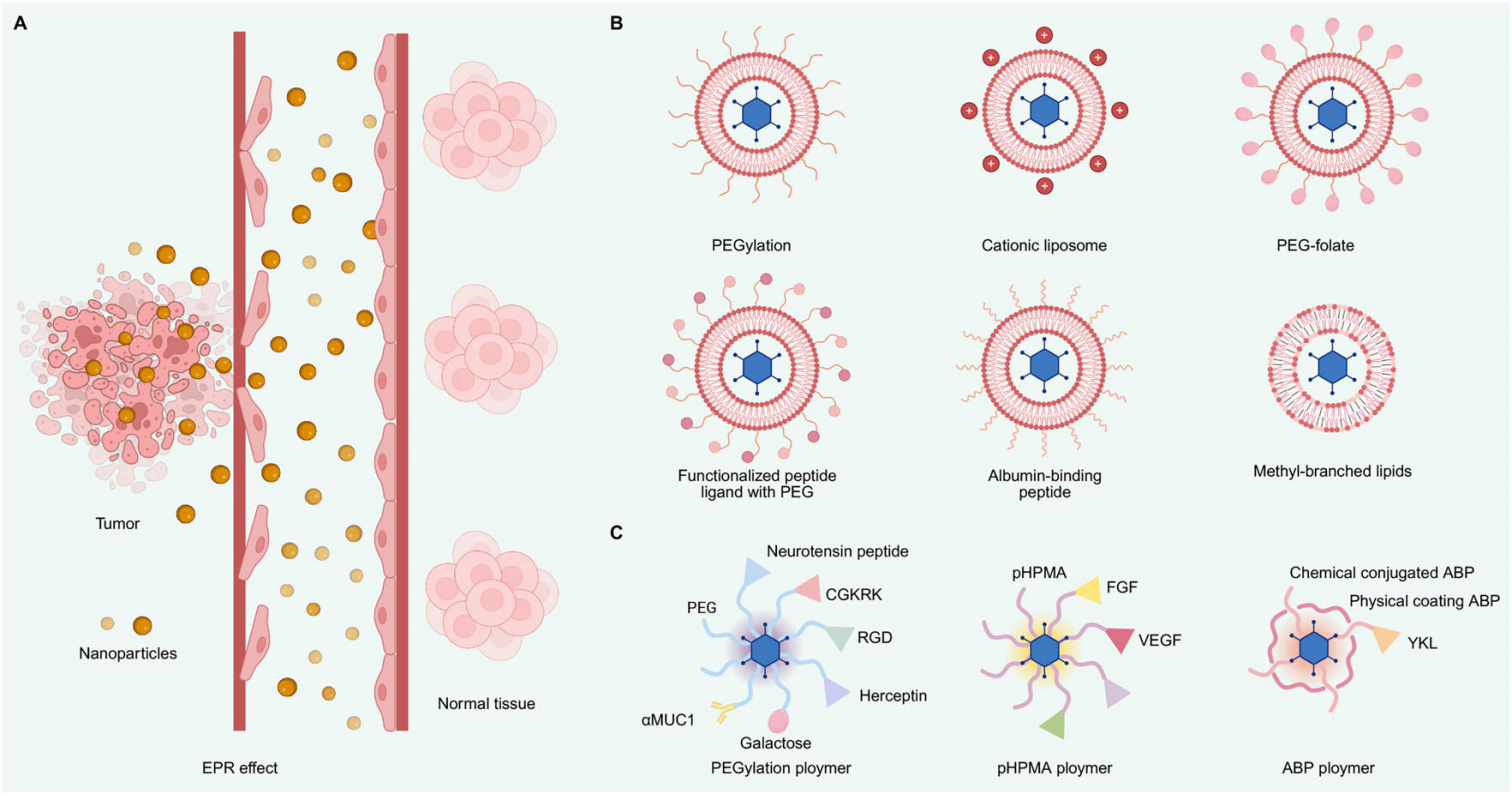
Nanoparticle‐based delivery systems enhance the systemic efficacy, tumor targeting, and intratumoral distribution of OVs. (A) Abnormal tumor vasculature and impaired lymphatic drainage allow nanoparticles to preferentially extravasate and accumulate within the tumor microenvironment through the EPR effect. This passive targeting results in increased intratumoral nanoparticle accumulation, improving viral bioavailability while reducing systemic exposure. (B) Several surface‐modification strategies were indicated: PEGylation shields viral particles from immune recognition and clearance, prolonging systemic circulation time; cationic liposomes facilitate efficient viral entry into tumor cells, circumventing antibody‐mediated neutralization in ascitic fluid; PEG–folate conjugates actively target tumor cells overexpressing folate receptors, significantly enhancing tumor‐specific uptake; peptide‐functionalized PEG–liposomes selectively bind tumor‐specific receptors such as integrins and growth factor receptors, improving cellular uptake; albumin‐binding peptides selectively enrich albumin on the liposomal surface to reduce immune recognition and enhance tumor‐specific accumulation; methyl‐branched lipids increase liposome membrane rigidity, stability, and controlled drug release, minimizing premature drug leakage and supporting sustained viral release within tumors. (C) Viral delivery is enhanced through synthetic polymer coatings that combine multiple functional modifications. PEGylation shields viral surface antigens to prolong circulation and reduce immune clearance, while conjugated targeting ligands (RGD, neurotensin, CGKRK, herceptin, galactose, or αMUC1) enable tumor‐specific accumulation. pHPMA polymers further enhance selectivity when conjugated with growth factors (VEGF, EGF) or integrin‐targeting peptides. Additionally, reducible ABP coatings provide controlled intracellular release under reductive conditions, promoting endosomal escape and intracellular viral release while minimizing systemic toxicity. These synergistic modifications collectively improve delivery efficiency through enhanced stability, targeted accumulation, and controlled activation. *Abbreviations*: OV, oncolytic virus; PEG, polyethylene glycol; VEGF, vascular endothelial growth factor; EGF, epidermal growth factor; RGD, arginine–glycine–aspartic acid peptide; CGKRK, tumor‐homing peptide; αMUC1, alpha mucin 1; pHPMA, poly(N‐(2‐hydroxypropyl)methacrylamide); ABP, amphiphilic block polymer; EPR, enhanced permeability and retention.

Among the nanoparticle systems, liposomes and polymeric nanoparticles represent the most clinically advanced systems for drug delivery [163]. Liposomes have proven biocompatibility and a high drug‐loading capacity. Clinically, pegylated liposomal doxorubicin (Doxil/Caelyx) is a landmark liposomal formulation with established therapeutic utility, including for acquired immunodeficiency syndrome‐related Kaposi's sarcoma and continues to be incorporated into treatment strategies for metastatic breast cancer [44, 164]. Similarly, polymeric nanoparticles offer stable drug encapsulation, adjustable release profiles, and versatile functionalization [165]. Notably, by leveraging TME features, particularly the enhanced permeability and retention (EPR) effect (Figure 6A), nanoparticles accumulate selectively in tumor tissues through leaky vasculature and impaired lymphatic drainage [166]. This targeted accumulation allows nanoparticles to penetrate the disorganized tumor vasculature and maintain therapeutic concentrations locally, thereby enhancing bioavailability and reducing systemic toxicity through controlled release, improved metabolic stability, and reduced clearance.

Given these advantages, nanoparticle‐mediated OV delivery has emerged as a promising strategy to enhance therapeutic efficacy while minimizing systemic toxicity. This section examines nanocarrier systems, such as liposomes and polymeric nanoparticles, and discusses their applications, limitations, and current challenges in OV delivery.

#### 4.3.1 Liposome‐Based Delivery

Liposomes are lipid‐based spherical nanoparticles with a phospholipid bilayer surrounding an aqueous core that encapsulates hydrophilic and lipophilic agents [167]. Since their introduction in the 1960s, liposomes have become essential drug delivery vehicles because of their biocompatibility, low immunogenicity, biodegradability, and controllable release properties [101, 102]. Their adaptable composition also supports the codelivery of multiple drugs or genes, enhancing therapeutic versatility and efficacy across applications, such as cancer, cardiovascular diseases, and inflammatory disorders [103].

Recently, liposomes have gained attention as OV carriers (Figure 6B). They protect OVs from neutralizing antibodies and immune clearance, thereby improving viral stability in circulation [104]. Surface modifications, such as PEGylation and ligand conjugation, extend the circulation time, reduce immune recognition, and promote tumor accumulation via the EPR effect [105, 106]. Yang et al. [168] reported that an EGFR‐targeted lipid envelope enhanced adenovirus delivery, increasing intratumoral accumulation while reducing liver sequestration. Similarly, cationic liposomes can shield reoviruses from ascites antibodies and promote entry via caveolin‐mediated endocytosis [169]. Other targeted strategies, such as folate‐modified or peptide‐functionalized liposomes, further enhance tumor‐selective uptake even in the absence of functional viral entry receptors [170]. Other targeted strategies, such as peptide‐functionalized liposomes bearing tumor‐homing sequences such as iRGD or EGFR‐binding peptides, have been shown to markedly enhance tumor‐selective uptake and accumulation by engaging overexpressed integrins or EGFRs, thereby improving precision drug delivery in preclinical cancer models [171].

Despite these promising results, liposomal delivery still faces challenges, such as immune‐mediated clearance, structural instability, and limited tumor penetration. To address these issues, strategies, such as surface modification with albumin‐binding peptides, have been used to modulate protein corona formation, reduce immune recognition, and enhance tumor‐specific uptake [107]. The incorporation of methyl‐branched lipids can improve membrane rigidity and stability, enabling sustained drug release [108]. Innovations, such as temperature‐sensitive liposomes, also allow for controlled drug release tailored to the TME [76, 109, 133]. Future research should focus on developing strategies that prolong the circulation time of liposomes, enhance their tumor‐targeting capability, and maintain viral stability to systematically improve targeted antitumor efficacy.

#### 4.3.2 Polymer‐Based Delivery

Polymers consisting of high‐molecular‐weight chains with repeating structural units are widely used in nanomedicine owing to their tunable physicochemical properties and synthetic versatility [110, 111, 112]. Their surface characteristics, whether hydrophilic or hydrophobic, can be tailored to improve biocompatibility and enhance drug delivery. Therapeutics can be stably incorporated via covalent bonding or physical interactions [113, 114]. Moreover, polymer‐based delivery achieves controlled drug release in response to specific triggers, such as pH changes, enzyme activity, or temperature, enabling targeted delivery with reduced off‐target effects [113, 115, 116]. Natural polymers include chitosan, collagen, cellulose, and glucans, while synthetic polymers include polylactic acid, polyglycolic acid, polybutyl cyanoacrylate, polyethyleneimine, and polyethylene glycol (PEG) [172, 173]. These materials vary in morphology, size, and biological properties, with selection depending on the drug characteristics. They are formulated as polymer–drug conjugates, biodegradable controlled‐release systems, and polymeric microparticles [174, 175]. Polymer–drug conjugates improve drug bioavailability and extend half‐life, whereas biodegradable systems enable sustained release over time. Polymeric microparticles can be engineered for enhanced targeting, stability, and controlled release, offering broad potential in oncology.

Polymer‐assisted delivery systems have shown encouraging progress in enhancing OV therapy (Figure 6C). Among OV platforms, adenoviruses have been the most extensively studied. PEGylation shields viral surface epitopes, which reduces neutralization by antibodies and inflammatory responses while increasing tumor‐specific accumulation relative to liver uptake. Tumor‐specific ligands provide an additional layer for precise targeting. For instance, bifunctional PEG–RGD and PEG–CGKRK guide viruses toward integrin‐enriched tumors and their associated vasculature. Similarly, PEG‐conjugated neurotensin receptor Type 1 peptides facilitate delivery to pancreatic tumors, whereas herceptin‐conjugated PEGylation enhances both systemic circulation and therapeutic efficacy in Her2/neupositive tumor models [176]. Poly‐N‐(2‐hydroxypropyl) methacrylamide decreases hepatic viral transduction. When conjugated with ligands, such as epidermal growth factor (EGF) or vascular endothelial growth factor (VEGF), it further enables active tumor‐targeted delivery. Agents, such as YKL‐1001‐ABP, an arginine‐grafted bio‐reducible polymer, have demonstrated selective cytolysis in hepatocellular carcinoma, prolonged circulation, and reduced IL‐6‐mediated hepatotoxicity [177, 178]. Additionally, cationic polymers with degradable linkers, such as poly (cystamine bisacrylamide–diaminohexane, CD) functionalized with PEG and cyclic RGD (CD–PEG–RGD), promote adenoviral entry via αvβ3/αvβ5 integrins independently of the coxsackie and adenovirus receptor. This enhances the cytopathic effects in integrin‐positive tumors, while sparing normal fibroblasts [179]. Similar modification strategies have been applied to other OVs. For HSV, galactose‐conjugated PEG improves selectivity toward hepatocellular carcinoma cells expressing asialoglycoprotein receptors, reduces off‐target replication in tissues such as the brain, and promotes CD8^+^ T and NK cell infiltration [180]. In the case of VV, mucin‐1‐targeted PEGylation (PEG–αMUC1) enhances pharmacokinetics and tumor selectivity in cancers with high mucin expression, such as pancreatic cancer [181].

Although polymer‐based delivery systems offer several advantages for OV transport, their clinical translation remains constrained by both biological and practical limitations. Compared with cell‐based or EV carriers, polymer nanoparticles often exhibit lower tumor accumulation and more limited tissue penetration, partly due to suboptimal drug‐loading capacity, inefficient viral release, and reduced transfection efficiency [117]. In addition, large‐scale manufacturing and quality control pose significant challenges, as polymer composition, batch‐to‐batch variability, and complex virus–polymer interactions complicate reproducibility and regulatory evaluation. Progress in polymer nanomaterials, including PEGylated systems, stimuli‐responsive smart polymers, and cationic systems, have partially addressed these limitations by improving circulation stability and controlled release. Preclinical studies combining polymer nanoparticles with OVs have demonstrated enhanced tumor targeting and reduced off‐target toxicity; however, their therapeutic efficacy, scalability, and safety profiles remain less mature than those of more biologically adaptive delivery platforms. Consequently, further optimization of formulation design, manufacturing standardization, and comparative efficacy assessments will be essential before polymer‐based OV delivery systems can achieve broader clinical applicability. While these delivery strategies demonstrate promising preclinical efficacy, their translation into safe and effective clinical therapies will require careful optimization to address challenges such as immunogenicity, tumor heterogeneity, and manufacturing constraints.

## 5 Combination Treatment Strategies

Refining OV delivery strategies helps partially evade neutralizing antibodies, enhance tumor accumulation, and reduce off‐target effects. Nevertheless, the immunosuppressive TME remains a key barrier to viral efficacy. Combining OVs with other therapies offers a promising approach to overcome this limitation by leveraging synergistic mechanisms to remodel the TME and amplify antitumor immunity.

### 5.1 Combination Strategies That Remodel the TME to Enhance Viral Efficacy

#### 5.1.1 Targeting ECM and Stromal Barriers

Excessive ECM deposition and the resulting increase in interstitial fluid pressure represent major physical barriers that limit the spread of OVs [182, 183]. Composed mainly of collagen, hyaluronic acid (HA), and glycosaminoglycans, the dense ECM restricts the lateral diffusion of viral particles, often trapping OVs near the injection site and hindering the deep penetration of tumor tissue after intratumoral delivery [184]. This structural impediment is further amplified by cancer‐associated fibroblasts (CAFs) that actively secrete ECM components. As key components of the TME, CAFs also drive tumor progression, angiogenesis, and immune evasion through the release of cytokines such as TGF‐β [185], VEGF [186], and CXCL12 [187]. A promising strategy for overcoming this challenge is to capitalize on the inherent susceptibility of CAFs to viral infections. Certain OVs selectively target specific CAF subsets [183]. Genetically modified OVs expressing bispecific T‐cell engagers against fibroblast activation protein (FAP) have been developed to recruit and activate T cells against FAP‐positive CAFs, thereby remodeling the immune microenvironment and enhancing intratumoral viral dissemination [188].

**FIGURE 7:**
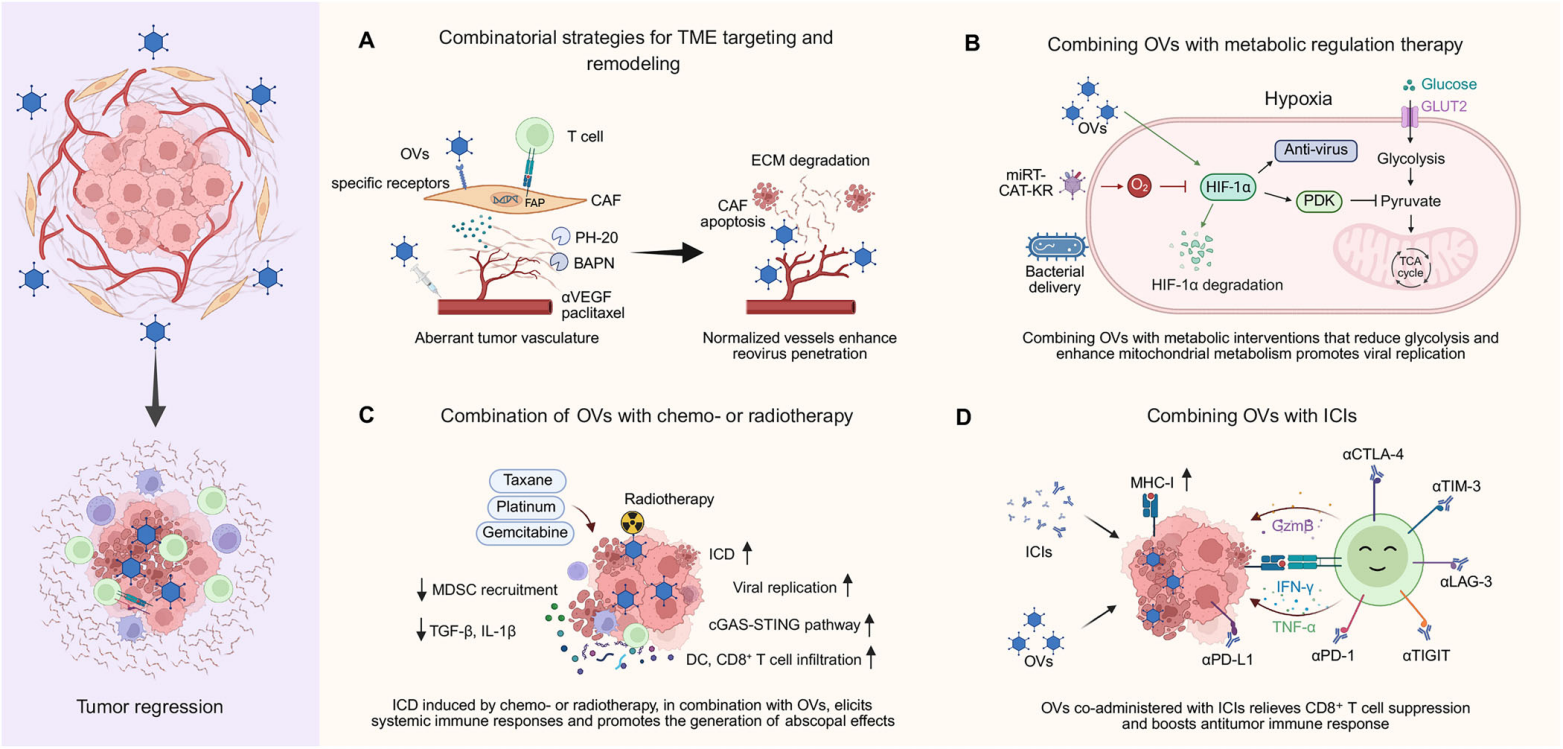
OVs combination therapies for enhanced cancer treatment. (A) OVs selectively infect CAFs expressing specific receptors, leading to CAF apoptosis and loss of tumor‐supportive functions. Physical barriers within the TME are mitigated through enzymatic or pharmacological approaches, including PH20 hyaluronidase‐mediated degradation of hyaluronic acid and BAPN‐mediated inhibition of collagen crosslinking, thereby reducing ECM density. Vascular normalization induced by anti‐VEGF therapy or low‐dose paclitaxel improves tumor perfusion, facilitating reovirus penetration and immune cell infiltration. (B) Hypoxia limits OVs replication and oncolytic activity through stabilization of HIF‐1α. HIF‐1α promotes glycolysis, suppresses mitochondrial TCA cycle activity and induces antiviral gene expression. Targeting these metabolic pathways can partially restore OVs therapeutic efficacy. In addition, hypoxia‐responsive delivery strategies, including oxygen‐generating systems (such as miRT–CAT–KR) and anaerobic bacterial carriers, enhance viral infectivity and immunogenicity within hypoxic tumor regions. (C) Chemotherapeutic agents, including taxanes, platinum‐based compounds, and gemcitabine, induce ICD and promote tumor antigen release. OVs infection amplifies ICD‐associated immune signaling and increases intratumoral antigen availability through productive viral replication. In combination, these treatments mitigate immunosuppressive features of the TME, thereby facilitating CD8^+^ T cell activation and infiltration. Radiotherapy further enhances antigen presentation and supports OVs replication. Together, these effects reinforce antitumor immunity and improve the efficacy of reovirus‐based virotherapy. (D) OVs triggers ICD, TAA release, and MHC‐I upregulation in tumor cells, facilitating CD8^+^ T cell activation. However, the TME often upregulates multiple inhibitory checkpoint molecules, leading to T cell exhaustion, impaired proliferation, and reduced cytotoxicity, which dampen reovirus‐elicited immune responses. Simultaneous blockade of these immunosuppressive pathways using multiple ICIs (e.g., anti‐PD‐1, anti‐PD‐L1, anti‐CTLA‐4, anti‐LAG‐3, anti‐TIM‐3, anti‐TIGIT) restores T cell functionality and promotes sustained antitumor responses. Reactivated T cells secrete elevated levels of effector cytokines (e.g., IFN‐γ, TNF‐α) and cytolytic molecules (e.g., GzmB), enhancing tumor cell clearance. *Abbreviations*: OV, oncolytic virus; CAF, cancer‐associated fibroblast; TME, tumor microenvironment; ECM, extracellular matrix; VEGF, vascular endothelial growth factor; HIF‐1α, hypoxia‐inducible factor‐1α; TCA, tricarboxylic acid; miRT–CAT–KR, microRNA‐targeted catalase system; ICD, immunogenic cell death; TAA, tumor‐associated antigen; MHC‐I, major histocompatibility complex Class I; ICI, immune checkpoint inhibitor; IFN‐γ, interferon‐gamma; TNF‐α, tumor necrosis factor‐alpha; GzmB, granzyme B.

ECM remodeling approaches were employed to improve the efficacy of OVs (Figure 7A). OVs engineered to express glycosylphosphatidylinositol‐anchored hyaluronidase PH20 have been shown to enhance viral distribution and promote M1 macrophage polarization in glioblastoma models by relieving HA‐mediated suppression of NF‐κB signaling [189]. Clinically, intravenously delivered PH20‐armed adenoviruses have demonstrated safety and favorable biodistribution across multiple tumor types [190]. Enzymatic modulation of the fibrotic stroma represents another effective approach for reducing intratumoral physical barriers. Collagenase and lysyl oxidase inhibitors such as β‐aminopropionitrile (BAPN) can degrade collagen or inhibit its crosslinking, thereby reducing matrix density and stiffness [191]. Combining these agents with OVs, such as collagenase in melanoma and sarcoma, or BAPN in tumors with dense stroma, enhances viral spread and tumor infection, thereby suppressing tumor progression and promoting immune cell infiltration.

Collectively, the dense ECM‐ and CAF‐rich stroma restricts OV dissemination and contributes to an immunosuppressive TME. Targeting ECM components or CAF populations may alleviate physical constraints on viral diffusion and shift the microenvironment toward a more permissive, proinflammatory state. Such stromal reprogramming has the potential to improve immune cell recruitment and enable deeper viral penetration into tumor cores, thereby potentially enhancing the overall viral efficacy.

#### 5.1.2 Normalizing Tumor Vasculature to Improve Viral Delivery

In addition to ECM‐targeting strategies, tumor vasculature improvement is another critical approach for enhancing the effectiveness of OVs (Figure 7A). Studies have indicated that short‐course, low‐dose VEGF blockade can induce a transient vascular normalization window, facilitating intratumoral delivery and activity of OVs, while minimizing excessive vessel pruning and therapy‐induced hypoxia [192]. Moreover, transient modulation of VEGF signaling creates a short‐lived vascular response window that improves intratumoral access and the therapeutic performance of systemically delivered OVs while avoiding the deleterious effects associated with sustained or high‐intensity antiangiogenic pressure [20, 44, 164, 193]. For instance, VEGF blockade with bevacizumab can transiently normalize tumor vasculature and improve functional perfusion and intratumoral access for systemically delivered therapeutics, which is mechanistically consistent with the enhanced intratumoral distribution of OVs and increased immune‐cell infiltration while mitigating therapy‐associated hypoxia when the antivascular effects are excessive [194, 195, 196, 197].

Collectively, vascular modulation represents a potential strategy for overcoming delivery‐related barriers in OVT. Precisely timed, dose‐controlled VEGF inhibition can induce temporary vascular normalization, improve intratumoral perfusion, and promote the entry of viral particles and immune effector cells while limiting hypoxia and detrimental vascular remodeling. Accordingly, the rational integration of vascular modulation with virotherapy may enhance viral dissemination, immune engagement, and overall therapeutic efficacy, particularly in the context of systemic OV administration.

#### 5.1.3 Metabolic Modulation to Overcome Hypoxia‐Associated Resistance

The hypoxic TME suppresses viral replication and oncolytic activity through several interconnected mechanisms. Hypoxia stabilizes hypoxia‐inducible factor‐1α (HIF‐1α), which initiates extensive metabolic reprogramming characterized by enhanced glycolytic flux and mitochondrial tricarboxylic‐acid cycle inhibition primarily through increased pyruvate dehydrogenase kinase. This shift reduces oxidative phosphorylation, ATP production, and overall cellular energy availability [198]. Simultaneously, HIF‐1α upregulates antiviral genes such as those encoding IFN‐I, pushing tumor cells into an antiviral state that further limits OV replication [199].

Emerging evidence supports hypoxia‐focused combination strategies, where pharmacological modulation of hypoxia and HIF‐1α‐associated pathways helps restore a permissive intracellular environment in oxygen‐deprived tumor regions, thereby improving oncolytic activity (Figure 7B) [198]. In parallel, hypoxia‐responsive regulatory elements, including HIF‐1α/HRE modules, have proven effective as hypoxia‐sensitive switches for transgene expression in engineered OVs, enabling the design of viruses adapted to hypoxic tumors [200]. Several indirect strategies have been developed to alleviate tumor hypoxia and potentiate OVT. Enhancing the intratumoral oxygen supply is a viable strategy for alleviating tumor hypoxia. The engineered adenovirus adv‐miRT‐CAT‐KR coexpresses catalase and the photosensitizer KillerRed, enabling local modulation of oxygen and redox homeostasis, which reduces HIF‐1α accumulation, promotes viral replication, and enhances antitumor immune responses [201]. These bacterial carriers have been shown to improve tumor accumulation after systemic administration, with targeting efficiencies approaching to those observed with intratumoral injections in preclinical models. Moreover, bacteria engineered to express pyranose oxidase can boost reactive oxygen species production and induce autophagy, thereby supporting viral replication, whereas PAMPs from both bacteria and viruses synergistically stimulate antitumor immune responses [202, 203, 204]. Tumor metabolic reprogramming provides an opportunity to overcome hypoxia‐associated resistance to virotherapy. Many solid tumors rely heavily on aerobic glycolysis and lactate fermentation to create an immunosuppressive metabolic milieu. Pharmacological attenuation of tumor glycolysis, mitigation of lactate‐driven immunosuppression, and metabolic reconditioning of the TME to favor antitumor immunity have been explored in preclinical studies as strategies to enhance the overall efficacy of OVT in a context‐dependent manner [205, 206, 207, 208].

Multitarget metabolic interventions aimed at enhancing mitochondrial metabolism and alleviating glycolytic dependence promote selective OV replication and reprogram the TME. Although these strategies offer promising directions for improving OVT in solid tumors, their safety and clinical efficacy require further validation.

### 5.2 Combination Strategies That Enhance Antitumor Immune Responses

Effective OVT depends on efficient viral infection and tumor cell lysis and induction of coordinated and durable antitumor immune responses. Although optimization of intratumoral conditions can improve viral delivery, replication, and spread, accumulating experimental and clinical evidence indicates that direct virus‐mediated cytotoxicity alone is insufficient to achieve sustained tumor control. Long‐term therapeutic benefits are closely linked to the ability of OVs to engage in host immune mechanisms that are capable of eliminating residual and disseminated malignant cells.

OV infection can initiate antitumor immunity by inducing ICD, promoting the release of TAAs and DAMPs, and activating innate immune‐sensing pathways that support antigen presentation and adaptive immune priming. However, in many solid tumors, these immune‐activating events are constrained by the immunosuppressive features of the TME, including immune checkpoint signaling, defective antigen presentation, and the accumulation of regulatory immune cell populations. Consequently, OV‐induced immune responses are often incomplete or transient and may fail to translate into sustained systemic antitumor immunity.

Accordingly, combination strategies designed to augment OV‐driven immune activation have become a central focus in OVT development. Combining OVs with therapies that enhance tumor immunogenicity or relieve immune suppression offers a rational strategy to strengthen and sustain antitumor immune responses. The following sections outline the key combinatorial strategies aimed at reinforcing host immunity and improving the depth and durability of OV‐based cancer therapies.

#### 5.2.1 Combination With Chemotherapy or Radiotherapy

Recent studies have demonstrated that combining OVs with conventional treatments, such as chemotherapy or radiotherapy, can substantially enhance their antitumor activity (Figure 7C). Chemotherapy exerts direct cytotoxic effects by inducing tumor cell death. In addition, many chemotherapeutic agents trigger ICD, promoting the release of tumor antigens and the activation of DCs. This therapy increases tumor antigenicity, modulates the TME, and reduces the presence of immunosuppressive cells such as MDSCs and Tregs [209]. Similarly, radiotherapy causes local tumor cell death through DNA damage while stimulating systemic immune responses. It enhances antigen presentation through the upregulation of MHC‐I and activates innate immune pathways such as cGAS–STING, thereby bridging innate and adaptive immunity [210]. Collectively, these mechanisms provide a strong rationale for combining OVs with chemotherapy or radiotherapy to improve therapeutic outcomes.

Emerging experimental and translational evidence indicates that rationally designed combinations of chemotherapy and virotherapy produce synergistic effects via multifaceted mechanisms. Chemotherapy‐remodeled TME enhances viral dissemination, immune infiltration, and therapeutic efficacy. The incorporation of OVs into cytotoxic chemotherapy regimens has been explored across multiple tumor types and delivery platforms. Intratumoral administration of T‐VEC in combination with standard neoadjuvant chemotherapy has demonstrated clinical feasibility and immune remodeling in triple‐negative breast cancer [211]. In addition to HSV‐based approaches, systemically delivered reoviruses have been evaluated in combination with taxane‐based chemotherapy in metastatic breast cancer, whereas VV (olvimulogene nanivacirepvec) has been used as an immunological priming agent prior to platinum‐based chemotherapy in ovarian cancer [212, 213]. Adenovirus‐based platforms (VCN‐01) have been investigated in combination with gemcitabine‐containing regimens for the treatment of pancreatic cancer [214]. Concurrently, OVs engineered to degrade ECM components, such as hyaluronidase‐armed VV or adenovirus VCN‐01, disrupt stromal barriers, facilitating both viral dissemination and chemotherapeutic penetration, particularly in desmoplastic tumors, such as pancreatic ductal adenocarcinoma [214, 215, 216]. Collectively, these results highlight the potential of chemotherapy as a combinatorial partner for OVs, as it can simultaneously modulate tumor cell susceptibility, stromal architecture, and antitumor immunity.

Radiotherapy is increasingly recognized as a rational combinatorial approach to OVT. Preclinical studies have indicated that combining OVs with radiotherapy improves tumor control, even at low viral doses, primarily by amplifying stress responses, apoptotic signaling, and bystander killing, rather than merely increasing viral infectivity [217]. In a mechanistic study using a T‐VEC‐like oncolytic HSV‐1 platform, the combination of OV and radiotherapy converted immunologically “cold” tumors into a CD8^+^ T‐cell‐inflamed phenotype, with IL‐1α identified as a key mediator. The addition of the PD‐1/PD‐L1 blockade further enhanced therapeutic efficacy and promoted systemic, immune‐mediated control of distant tumors, supporting a radio‐viro‐immunotherapy strategy for ICI‐refractory disease [217]. Earlier Phase I experience with intratumoral reovirus combined with fractionated radiotherapy also suggests acceptable tolerability and provides a proof‐of‐concept for flexible treatment sequencing, which is consistent with the observation that certain OV platforms remain active under ionizing radiation [218]. Overall, current evidence suggests that radiotherapy can act as both a cytotoxic and immunological amplifier of OVT, with treatment timing and dose fractionation likely influencing the extent of synergy and systemic response.

However, several challenges remain to be resolved. The optimal treatment parameters, including dosage, timing, and sequencing, must be clearly established through rigorous clinical studies. Furthermore, variations in tumor types and immune microenvironments emphasize the need for personalized therapeutic strategies. These approaches are likely to be informed by reliable biomarkers such as RAS activation status or immune profiling. Despite these complexities, OVs hold considerable promise as part of multimodal cancer therapy strategies. When thoughtfully integrated with chemotherapy or radiotherapy, the treatment efficacy may be significantly enhanced. Continued translational and clinical research is essential to address these current limitations and ultimately improve patient outcomes.

#### 5.2.2 Combining OVs With ICIs

Theoretically, OVs induce ICD, leading to the release of TAAs and the initiation of antitumor immune responses. However, the immunosuppressive TME marked by inhibitory pathways, such as PD‐1/PD‐L1 and CTLA‐4, impairs T cell function and significantly diminishes the effectiveness of this immune activation. T lymphocytes play a crucial role in immune surveillance by recognizing and eliminating pathogens and other abnormal cells. Immune checkpoints normally help maintain immune homeostasis; however, tumors frequently co‐opt these pathways to suppress T‐cell activity and evade immune destruction. ICIs counteract this suppression by blocking inhibitory signals, thus restoring T‐cell function and enabling effective immune responses against tumor cells. The currently approved ICIs primarily target CTLA‐4, PD‐1, and PD‐L1, which are key regulators of T‐cell exhaustion and dysfunction [219, 220]. CTLA‐4 primarily modulates early T‐cell activation, whereas PD‐1/PD‐L1 signaling dampens effector T‐cell function in peripheral tissues later in the immune response [221]. Upon PD‐1 engagement, cytokine production, encompassing TNF, IL‐2, and IFN‐γ, is reduced, antiapoptotic genes are downregulated, and T‐cell proliferation and cytotoxicity are inhibited. Additional checkpoints such as TIM3, LAG3, and TIGIT also contribute to immune suppression [164], although their full roles remain under active investigation.

Mechanistically, ICIs align well with OV therapy by restoring effector T‐cell proliferation, cytokine production, and cytotoxicity, thereby converting the transient inflammatory response triggered by OVs into sustained antitumor immunity (Figure 7D) [7]. Evidence supporting this synergy now spans diverse OVs, including HSV‐1 and multiple adenoviral systems and has been extended to reoviruses in clinically relevant studies [222, 223, 224, 225]. In recurrent glioblastoma, intratumoral DNX‐2401 combined with systemic pembrolizumab demonstrated clinical activity, with immune correlates indicating therapy‐induced remodeling of the TME [222]. In patients with anti‐PD‐1‐resistant melanoma, the adenovirus ONCOS‐102 plus pembrolizumab was well tolerated, increased intratumoral cytotoxic CD8^+^ T cell infiltration, and produced objective responses in a subset of patients [223]. Similarly, engineered HSV‐1 (RP1) combined with nivolumab induced deep and durable systemic responses in melanoma refractory to PD1 blockade, further validating the clinical potential of the OV–ICI combination [224].

Across these studies, common immunological themes have emerged, including enhanced innate sensing and Type I IFN signaling, improved antigen processing and presentation, expansion of tumor‐infiltrating CD8^+^ T cells, and a reduction in immunosuppressive myeloid and regulatory T‑cell populations. Together, these shifts reflect an OV‐driven transition toward a more immune‐permissive TME. Importantly, such immunomodulatory properties are particularly relevant for tumors characterized by poor baseline immune infiltration and limited responsiveness to immune checkpoint blockades, such as hepatocellular carcinoma. In these settings, OVs may serve as immune‐priming agents that disrupt the suppressive TME, enhance immune cell recruitment, and restore antitumor immune competence. Sequential or rationally timed administration of ICIs following OV‐mediated immune reconditioning may represent a promising therapeutic strategy to overcome primary resistance to checkpoint inhibition.

#### 5.2.3 Interviral Combination Strategies in OVT

The fusion‐associated small transmembrane (FAST) protein, encoded by the reovirus, promotes syncytium formation and induces ICD, thereby enhancing TAA release and antitumor immune activation [226, 227, 228]. Capitalizing on this mechanism, a recombinant VSV expressing the FAST protein (VSV–p14) has been developed. In murine models of breast and metastatic colorectal cancers, VSV–p14 demonstrated superior tumor control and prolonged survival compared with that of conventional VSV, accompanied by robust activation of CD4^+^ T cells, CD8^+^ T cells, and NK cells [229]; however, these findings are based solely on preclinical models, and their relevance in humans remains to be established. Sequential administration of different OVs also demonstrated synergistic potential. For instance, VV can initially suppress local antiviral responses, creating a permissive environment for subsequent VSV infection and enhancing tumor cell killing [230]. In another approach, intratumoral administration of reovirus, followed by intravenous administration of VSV engineered to express tumor antigens, generated complementary Th1 and Th17 responses [196, 231]. When this combined viral regimen was paired with αPD‐1 therapy, it significantly improved survival and achieved complete tumor regression with long‐term tumor‐free survival in all treated mice under experimental conditions [232].

Each OV platform exhibits distinct biological characteristics. Adenovirus and HSV can be extensively engineered to enhance immunogenicity. VV potently suppresses innate immunity, reovirus triggers robust early innate immune activation, and VSV exhibits rapid and potent oncolysis but remains sensitive to IFN‐mediated antiviral responses [224, 230, 232]. Therefore, the rational combination of OVs based on their complementary properties presents a promising strategy for overcoming the inherent limitations of single‐virus therapies and improving therapeutic outcomes in solid tumors. However, this multiviral approach introduces additional complexities. Determining the optimal sequence, timing, and dosage of each virus is critical and may vary depending on the tumor type and immune context. Potential safety concerns, including enhanced inflammatory responses and unexpected viral interactions, require careful preclinical evaluation. Furthermore, the development of scalable manufacturing processes and clinically feasible administration schedules for combination virotherapy remains challenging. Despite these challenges, the strategic combination of OVs provides a promising approach to achieving more potent and durable antitumor immunity, warranting further investigation in translational and clinical studies.

## 6 Clinical Development Landscape of OV‐Based Therapies

An analysis of records from ClinicalTrials.gov revealed that 171 clinical trials evaluating OV‐based therapies have been initiated worldwide. Among these, 62 trials (36%) were completed (Table 2), whereas 109 trials (64%) are either ongoing or remain in other active stages of development (Table 3). The current clinical landscape involves a diverse array of OVs, including adenoviruses, HSV‐1, VV, reovirus, MV, poliovirus, coxsackievirus, parvovirus, and VSV. This diversity reflects the continued exploration of distinct viral properties and therapeutic mechanisms.

**TABLE 2:** Completed clinical trials of OV‐based therapy.

NCT number	Types of tumors	Phases	Types of viruses	Combined treatment	Injection strategy
NCT05393440	Glioma, astrocytoma, glioblastoma	1,2	Herpes simplex virus Type 2	∖	i.t.
NCT06562621	Glioblastoma multiforme, gliomas, malignant	1,2	Herpes simplex virus Type 1	∖	i.t.
NCT03072134	Glioma, anaplastic astrocytoma, anaplastic oligodendroglioma, glioblastoma multiforme, astrocytoma	1	Oncolytic adenovirus	∖	i.t.
NCT04771676	Refractory malignant ascites	2	Oncolytic adenovirus	∖	i.p.
NCT01846091	Head and neck squamous cell carcinoma, breast carcinoma	1	Oncolytic measles virus	∖	i.t.
NCT02197169	Glioblastoma, gliosarcoma	1	Oncolytic adenovirus	Interferon‐gamma	i.t.
NCT03178032	Brainstem glioma, neoadjuvant therapy	1	Oncolytic virus	∖	i.t.
NCT03605719	Recurrent plasma cell myeloma	1	Pelareorep (reovirus)	Carfilzomib, dexamethasone, nivolumab	i.v.
NCT01503177	Recurrent malignant mesothelioma	1	Oncolytic measles virus	∖	i.t.
NCT02192775	Multiple myeloma	2	Oncolytic measles virus	Cyclophosphamide	i.v.
NCT02428036	Malignant melanoma and squamous cell carcinoma of the skin	1	HSV‐1	∖	i.t.
NCT03004183	Metastatic non‐small cell lung cancer, metastatic triple‐negative breast cancer	2	Oncolytic adenovirus, Herpes simplex virus	Valacyclovir, pembrolizumab, radiation	i.t.
NCT02053220	Resectable colon cancer, resectable non‐small cell lung cancer, resectable bladder cancer, resectable renal cell carcinoma	1	Oncolytic adenovirus	∖	i.t./i.v.
NCT02759588	Ovarian cancer, peritoneal carcinomatosis, fallopian tube cancer	1,2	Oncolytic vaccinia virus	∖	i.p.
NCT00408590	Ovarian cancer, primary peritoneal cavity cancer	1	Oncolytic measles virus	∖	i.p.
NCT03043391	Malignant glioma, anaplastic astrocytoma, anaplastic oligoastrocytoma, anaplastic oligodendroglioma, glioblastoma, gliosarcoma, atypical teratoid/rhabdoid tumor of brain, medulloblastoma, ependymoma, pleomorphic xanthoastrocytoma of brain, embryonal tumor of brain	1	Polio/rhinovirus recombinant	∖	i.t.
NCT02028442	Solid tumors of epithelial origin, metastatic colorectal cancer, metastatic bladder cancer	1,2	Oncolytic adenovirus	∖	i.v.
NCT02028117	Recurrent platinum‐resistant ovarian cancer	1	Oncolytic adenovirus	∖	i.p./i.v.
NCT02365818	Bladder cancer	2	Oncolytic adenovirus	∖	i.v.
NCT00794131	Prostate cancer	1	Oncolytic vaccinia virus	∖	i.v.
NCT03954067	Metastatic cancer, solid tumors, advanced cancer	1	Herpes simplex virus Type 1	Pembrolizumab	i.t.
NCT02045602	Locally advanced solid tumors, metastatic solid tumors, pancreatic adenocarcinoma	1	Oncolytic adenovirus	gemcitabine, abraxane	i.v.
NCT00450814	Recurrent plasma cell myeloma, refractory plasma cell myeloma	1,2	Oncolytic measles virus	Cyclophosphamide	i.v.
NCT01443260	Peritoneal carcinomatosis	1,2	Vaccinia virus	∖	i.p.
NCT01956734	Glioblastoma multiforme, recurrent tumor	1	Oncolytic adenovirus	Temozolomide	i.t.
NCT00429312	Melanoma	1,2	Vaccinia virus	GM‐CSF	i.t.
NCT04217473	Metastatic melanoma	1	Oncolytic adenovirus	∖	i.v./i.t.
NCT03852511	Metastatic cancer, epithelial tumor	1	Oncolytic adenovirus	∖	i.v.
NCT00554372	Carcinoma, hepatocellular	2	Vaccinia virus	∖	i.t.
NCT03206073	Colorectal cancer, colorectal carcinoma, colorectal adenocarcinoma, colorectal neoplasms	1,2	Vaccinia virus	Durvalumab, tremelimumab	i.v.
NCT00629759	Neoplasms, liver	1	Vaccinia virus	∖	i.t.
NCT02798406	Glioma, glioblastoma, gliosarcoma, neuroepithelial, neuroectodermal tumors	2	Oncolytic adenovirus	Pembrolizumab	i.t.
NCT03408587	Uveal melanoma	1	Coxsackievirus	Ipilimumab	i.v.
NCT02045589	Pancreatic adenocarcinoma, metastatic pancreatic adenocarcinoma	1	Oncolytic adenovirus	Gemcitabine, abraxane	i.t.
NCT01598129	Malignant solid tumor	1	Oncolytic adenovirus	∖	i.v./i.t.
NCT01584284	Cancer of head and neck	1	Vaccinia virus	∖	i.v.
NCT01227551	Malignant melanoma	2	Coxsackievirus	∖	i.t.
NCT02879760	Non‐small cell lung cancer	1	Vaccinia virus, oncolytic adenovirus	Pembrolizumab	i.v.
NCT02562755	Hepatocellular carcinoma	3	Vaccinia virus	Sorafenib	i.t.
NCT01017185	Refractory head and neck cancer, squamous cell carcinoma, skin, carcinoma of the breast, malignant melanoma	1	Herpes simplex virus Type 1	＼	i.t.
NCT03663712	Peritoneal surface malignancy	1	Herpes simplex virus Type 1	∖	i.p.
NCT03916510	Locally advanced rectal cancer	1	Oncolytic adenovirus	Capecitabine, radiotherapy	∖
NCT00438009	Melanoma	1	Coxsackievirus	∖	i.t.
NCT03889275	Malignant melanoma	1	Herpes simplex virus Type 1	Durvalumab	i.v.
NCT00503295	Osteosarcoma, Ewing sarcoma family tumors, malignant fibrous histiocytoma, sarcoma, synovial, fibrosarcoma, leiomyosarcoma	2	Reovirus	∖	i.v.
NCT03153085	Melanoma	2	Herpes simplex virus Type 1	Ipilimumab	i.v.
NCT03171493	Urothelial carcinoma	1	Oncolytic measles virus	∖	i.v.
NCT01301430	Glioblastoma multiforme	1,2	Parvovirus	∖	i.v./i.t.
NCT02272855	Malignant melanoma	2	Herpes simplex virus Type 1	Ipilimumab	i.v.
NCT00289016	Melanoma	2	Herpes simplex virus Type 1		i.t.
NCT05673811	Pancreatic adenocarcinoma, metastatic	2	Oncolytic adenovirus	Nab‐paclitaxel, gemcitabine	i.v.
NCT00625456	Melanoma, lung cancer, renal cell carcinoma, squamous cell carcinoma of the head and neck	1	Vaccinia virus	＼	i.v.
NCT00028158	Malignant pleural mesothelioma	1,2	Herpes simplex virus Type 1	∖	i.t.
NCT02457845	Supratentorial neoplasms, malignant, malignant glioma, glioblastoma, anaplastic astrocytoma, PNET, cerebral primitive neuroectodermal tumor, embryonal tumor	1	Herpes simplex virus Type 1	∖	i.t.
NCT02620423	Pancreatic adenocarcinoma	1	Reovirus	Chemotherapy, gemcitabine, irinotecan, leucovorin, 5‐fluorouracil, pembrolizumab	i.v.
NCT01394939	Colorectal carcinoma	1,2	Vaccinia virus	Irinotecan	i.v.
NCT02700230	Metastatic malignant peripheral nerve sheath tumor, recurrent malignant peripheral nerve sheath tumor	1	Oncolytic measles virus	∖	i.t.
NCT01169584	Neuroblastoma, rhabdomyosarcoma, lymphoma, Wilm's tumor, Ewing's sarcoma	1	Vaccinia virus	∖	i.t.
NCT01274624	KRAS mutant metastatic colorectal cancer	1	Reovirus	Irinotecan, leucovorin, fluorouracil, bevacizumab	i.v.
NCT00528684	Malignant glioma	1	Reovirus	∖	i.t.
NCT01387555	Hepatocellular carcinoma	2	Herpes simplex virus Type 1	∖	∖
NCT02977156	Metastatic tumor, advanced tumor	1	Vaccinia virus	Ipilimumab	i.t.

*Data sources*: ClinicalTrials.gov.

**TABLE 3:** Ongoing and active clinical development of OV‐based therapies.

NCT Number	Types of tumors	Phases	Types of viruses	Combined treatment	Injection strategy
NCT05860374	Sarcoma, breast cancer, pancreatic cancer, colorectal cancer, gastric cancer, liver cancer, lung cancer, gynecologic cancer	1	Herpes simplex virus Type 1	∖	i.t./i.p.
NCT05961111	Sarcoma, digestive cancer, breast cancer, lung cancer, brain cancer, melanoma, gynecologic cancer, head and neck cancer, kidney cancer	1	Herpes simplex virus Type 1	∖	i.t.//i.p.
NCT05830240	Head and neck cancer, esophageal cancer, otorhinolaryngologic neoplasms, ear cancer, nose cancer, laryngeal cancer, pharyngeal cancer	1	Herpes simplex virus Type 1	∖	i.t.
NCT06171282	Osteosarcoma, sarcoma, soft tissue sarcoma, bone tumor	1	Herpes simplex virus Type 1	∖	i.t.
NCT05812677	Cervical cancer, endometrial cancer, advanced cancer	1	Herpes simplex virus Type 1	∖	i.t./i.p.
NCT05851456	Osteosarcoma, sarcoma, sarcoma, soft tissue, bone tumor	1	Herpes simplex virus Type 1	∖	i.t.
NCT06758544	Pancreatic cancer	1,2	Oncolytic alphavirus	Chemotherapy	∖
NCT06910657	Colorectal cancer, pancreatic cancer, melanoma, ovarian cancer, gastric cancer, esophageal cancer, hepatocellular carcinoma, renal cell carcinoma, breast cancer, sarcoma, bladder cancer, lung cancer, prostate cancer, cervical cancers, head and neck cancers, adrenal gland tumors	1	Vaccinia virus	∖	i.v.
NCT06346041	Malignant solid tumors	1	IDOV‐SAFETM	∖	i.v.
NCT04349436	Cutaneous squamous cell carcinoma, Merkel cell carcinoma, basal cell carcinoma, melanoma	1,2	Herpes simplex virus Type 1	∖	i.t.
NCT07006077	Advanced pancreatic cancer	2	Vaccinia virus	∖	i.t.
NCT07185243	Advanced solid tumors	NA	Vaccinia virus	Tislelizumab	i.t.
NCT07061704	Esophageal cancer	1,2	Not provided	Chemotherapy, PD‐1 inhibitor	i.t.
NCT06380309	Malignant digestive system tumors	1	DOV‐SAFETM	Toripalimab, fruquintinib	i.v.
NCT06031636	Malignant pleural mesothelioma, advanced		Oncolytic adenovirus	PD‐1 inhibitors	i.t./i.p.
NCT03866525	Gastrointestinal cancer	1,2	Herpes simplex virus Type 2	Irinotecan, HX008	i.t.
NCT06552598	Cervical cancer	1	Oncolytic adenovirus	＼	i.t.
NCT06632964	Nonmuscle invasive bladder cancer	1	Oncolytic adenovirus	∖	i.v.
NCT07018518	Hepatocellular carcinoma, adult	1	Newcastle disease virus	Anti‐PD1	i.t.
NCT06883149	Solid tumor, adult	1	Newcastle disease virus	Anti‐PD1	i.t.
NCT05235074	Central nervous system tumors	1,2	Herpes simplex virus Type 2	∖	i.t.
NCT05232136	Nonmuscle‐invasive bladder cancer	1,2	Herpes simplex virus Type 2	∖	i.v.
NCT06718946	Advanced malignant solid tumor	1	IDOV‐SAFTM	∖	i.v.
NCT04695327	Advanced solid tumor	1	Oncolytic adenovirus	∖	i.t.
NCT05346484	Advanced solid tumor, cholangiocarcinoma, bile duct cancer	1	Chimeric orthopoxvirus	Modified FOLFOX, pembrolizumab	i.t./i.v.
NCT05717712	Diffuse intrinsic pontine glioma	1	Oncolytic adenovirus	∖	i.t.
NCT05717699	Diffuse intrinsic pontine glioma	1	Oncolytic adenovirus	∖	i.t.
NCT06581406	Metastatic uveal melanoma	2,3	Herpes simplex virus Type 1	Ipilimumab, nivolumab	∖
NCT06508307	Sarcoma, cervical cancer, colon cancer, lung cancer, ovarian cancer, pancreatic cancer, hepatocellular carcinoma, breast cancer, gastric cancer	1	Vaccinia virus	∖	i.t.
NCT04386967	Melanoma	1,2	Herpes simplex virus Type 2	Keytruda, anti‐PD‐1 antibody	i.t.
NCT04616443	Melanoma	1,2	Herpes simplex virus type	Keytruda	i.t.
NCT05868707	Melanoma	3	Herpes simplex virus Type 2	∖	i.t.
NCT05698459	Advanced liver cancer	1	Herpes simplex virus Type 2	∖	i.t.
NCT06618235	Ovarian cancer	1,2	THEO‐260	∖	∖
NCT06585527	Glioblastoma multiforme, glioma, malignant	1	Oncolytic adenovirus	∖	i.t.
NCT06368921	Solid tumor	1	Oncolytic alphavirus	∖	i.t.
NCT06961786	Metastatic melanoma	1	Oncolytic adenovirus	TILs, cyclophosphamide, fludarabine	i.t.
NCT06757153	Glioblastoma	1	Oncolytic adenovirus	∖	＼
NCT06126744	Recurrent high‐grade glioma	1	Herpes simplex virus Type 1	∖	i.t.
NCT05427487	Colorectal cancer, gastric cancer, ovarian cancer	1	IVX037	Sintilimab	i.t.
NCT05076760	Solid tumor, advanced cancer, metastatic cancer, on‐small cell lung cancer, cutaneous squamous cell carcinoma, Merkel cell carcinoma, melanoma, pancreatic cancer, triple negative breast cancer, head and neck cancer	1	Oncolytic adenovirus	Nivolumab, docetaxel	i.t.
NCT06660056	High‐grade gliomas	1	Vaccinia virus	∖	i.t.
NCT06444815	Solid tumor, adult, microsatellite stable colorectal cancer, head and neck squamous cell carcinoma, cervical cancer, kidney cancer, renal cell carcinoma, melanoma Stage IV, Merkel cell carcinoma of skin, mesothelioma, on‐small cell lung cancer	1	Vaccinia virus	Pembrolizumab	i.t.
NCT06216938	Melanoma	1	Herpes simplex virus Type 1	∖	i.t.
NCT05684731	Ovarian cancer	1	Vaccinia virus	Chemotherapy	i.p.
NCT04521764	Anatomic Stage IV breast cancer AJCC v8, invasive breast carcinoma, metastatic breast adenocarcinoma, recurrent breast carcinoma, Stage IV breast cancer AJCC v6 and v7	1	Oncolytic measles virus	∖	i.t.
NCT04673942	Sarcoma, sarcoma, soft tissue, chondrosarcoma	2	Oncolytic virus	Checkpoint inhibitor	i.t.
NCT06311578	Advanced solid tumors	1	JNJ‐87704916	Cetrelimab	i.t.
NCT07231458	Advanced solid tumors	1	ABX‐001	Pembrolizumab	i.v.
NCT06826313	Solid tumors	1	Oncolytic alphavirus	∖	i.v.
NCT05733598	Hepatocellular carcinoma, biliary tract cancer	2	Herpes simplex virus Type 1	Bevacizumab, atezolizumab	i.t.
NCT06265025	Head and neck cancer, malignant melanoma, colorectal cancer, renal cell carcinoma, cervical cancer, breast cancer	1,2	Oncolytic adenovirus	Pembrolizumab	i.t.
NCT05281471	Platinum‐resistant ovarian cancer, platinum‐refractory ovarian cancer, fallopian tube cancer, primary peritoneal cancer, high‐grade serous ovarian cancer, endometrioid ovarian cancer, ovarian clear cell carcinoma	3	Vaccinia virus	Platinum chemotherapy, nonplatinum chemotherapy, bevacizumab	i.p.
NCT03740256	Bladder cancer, head and neck squamous cell carcinoma, cancer of the salivary gland, lung cancer, breast cancer, gastric cancer, esophageal cancer, colorectal cancer, pancreatic adenocarcinoma, solid tumor	1	Oncolytic adenovirus	∖	i.t.
NCT05124002	Cholangiocarcinoma, intrahepatic	4	Oncolytic adenovirus	Oxaliplatin, leucovorin calcium	i.t.
NCT07128914	Solid tumor malignancies	1	Oncolytic vaccinia virus	∖	i.t.
NCT06463665	Advanced nonsquamous non‐small‐cell lung cancer, advanced squamous non‐small cell lung carcinoma, metastatic nonsquamous non‐small cell lung cancer, metastatic squamous non‐small cell lung carcinoma, on‐small cell lung cancer, non‐small cell lung cancer Stage Iino‐small cell lung cancer Stage IV, non‐small cell lung cancer recurrent	2	Oncolytic vaccinia virus	Platinum chemotherapy, nonplatinum chemotherapy, physician's choice of immune checkpoint inhibitor, docetaxel	i.v.
NCT06046742	Advanced or metastatic solid tumors	1	Oncolytic alphavirus	∖	i.v.
NCT07001592	Gastric neoplasms, esophageal cancer, liver cancer, liver metastasis, MSS‐CRC, MSS, gastric adenocarcinoma, peritoneal cancer, peritoneal carcinoma, peritoneal metastases, MSI‐H, gastric cancer	1	Vaccinia virus	∖	i.t.
NCT03152318	Malignant glioma of brain, astrocytoma, malignant astrocytoma, oligodendroglioma, anaplastic oligodendroglioma of brain (diagnosis), mixed oligo‐astrocytoma, ependymoma, ganglioglioma, pylocytic/pylomyxoid astrocytoma, brain tumor, glioma, brain cancer, glioblastoma, glioblastoma multiforme	1	Herpes simplex virus Type 1	Cyclophosphamide	i.t.
NCT05139056	Currently only enrolling glioblastoma patients at first recurrence	1	Oncolytic adenovirus	∖	i.t.
NCT06889493	Neuroendocrine carcinoma, neuroendocrine tumors	1	Oncolytic picornavirus	Nivolumab, ipilimumab	i.t.
NCT05271318	Ovarian cancer fallopian tube carcinoma, peritoneal carcinoma	1,2	Oncolytic adenovirus	Pegylated liposomal doxorubicin, pembrolizumab	∖
NCT03911388	Neuroepithelial, neoplasms	1	Herpes simplex virus Type 1	∖	i.t.
NCT06508463	T cell lymphoma	1	Vesicular stomatitis virus	Cemiplimab, ipilimumab	i.v.
NCT07260591	Melanoma, breast cancer, lung cancer	1	Vesicular stomatitis virus	∖	i.v./i.t.
NCT03017820	B‐cell non‐Hodgkin lymphoma, histiocytic and dendritic cell neoplasm, recurrent adult acute myeloid leukemia, recurrent anaplastic large cell lymphoma, recurrent angioimmunoblastic T‐cell lymphoma, recurrent mycosis fungoides, recurrent plasma cell myeloma, recurrent primary cutaneous T‐cell non‐Hodgkin lymphoma, recurrent T‐cell non‐Hodgkin lymphoma, refractory acute myeloid leukemia, refractory anaplastic large cell lymphoma, refractory angioimmunoblastic T‐cell lymphoma, refractory mycosis fungoides, refractory peripheral T‐cell lymphoma, not otherwise specified, refractory plasma cell myeloma, refractory primary cutaneous T‐cell non‐Hodgkin lymphoma, refractory T‐cell non‐Hodgkin lymphoma	1	Vesicular stomatitis virus	Ruxolitinib, cyclophosphamide	i.v.
NCT05914376	Advanced solid tumors	1	Vaccinia virus	∖	i.t.
NCT05081492	Breast cancer	1	Orthopoxvirus	∖	i.t.
NCT02705196	Pancreatic cancer	1,2	Oncolytic adenovirus	Gemcitabine, nab‐paclitaxel, atezolizumab	i.t.
NCT05051696	Genital neoplasms, female	2	Oncolytic adenovirus	Radiotherapy	i.t.
NCT04050436	Cutaneous squamous cell carcinoma	2	Herpes simplex virus Type 1	Cemiplimab	i.t.
NCT05733611	Refractory metastatic colorectal cancer	2	Herpes simplex virus Type 1	Atezolizumab, bevacizumab	i.t.
NCT05222932	Melanoma, head and neck squamous cell carcinoma	1	Oncolytic adenovirus	Avelumab	∖
NCT04445844	Breast cancer	2	Reovirus	Pelareorep, retifanlimab	i.v.
NCT02285816	Advanced/metastatic solid tumors	1,2	Oncolytic adenovirus	∖	i.v.
NCT03657576	Glioblastoma multiforme of brain, anaplastic astrocytoma of brain, gliosarcoma of brain	1	Herpes simplex virus Type 1	∖	i.t.
NCT03294083	Renal cell carcinoma	1,2	Vaccinia Virus	Cemiplimad	i.v.
NCT00092222	Lymphoproliferative disorder	2	Oncolytic herpesvirus	Etoposide, interferon‐alpha, rituximab, zidovudine, liposomal doxorubicin, bortezomib, valganciclovir, doxorubicin, vincristine, cyclophosphamide, filgrastim (G‐CSF), prednisone, sirolimus	i.t.
NCT02062827	Recurrent glioblastoma multiforme, progressive glioblastoma multiforme, anaplastic astrocytoma, or gliosarcoma	1	Herpes simplex virus Type 1	∖	i.t.
NCT03252808	Pancreatic cancer	1	Herpes simplex virus Type 1	Gemcitabine, nab‐paclitaxel, TS‐1	i.t.
NCT04735978	Advanced solid tumor	1	Herpes simplex virus Type 1	Nivolumab	i.t.
NCT06063317	Solid tumor, adult	1	Orthopoxvirus	Blinatumomab, hydroxyurea	i.t.
NCT03647163	Non‐small cell lung cancer, neuroendocrine carcinoma, renal cell carcinoma	1,2	Vesicular stomatitis virus	Pembrolizumab, ipilimumab, nivolumab	i.v.
NCT02068794	Fallopian tube clear cell adenocarcinoma, fallopian tube endometrioid adenocarcinoma, fallopian tube mucinous adenocarcinoma, fallopian tube serous adenocarcinoma, fallopian tube transitional cell carcinoma, fallopian tube undifferentiated carcinoma, malignant ovarian Brenner tumor, ovarian clear cell adenocarcinoma, ovarian endometrioid adenocarcinoma, ovarian mucinous adenocarcinoma, ovarian seromucinous carcinoma, ovarian serous adenocarcinoma, ovarian transitional cell carcinoma, ovarian undifferentiated carcinoma, primary peritoneal serous adenocarcinoma, recurrent fallopian tube carcinoma, recurrent ovarian carcinoma, recurrent primary peritoneal carcinoma	1,2	Oncolytic measles virus	∖	i.p.
NCT04215146	Breast cancer metastatic	2	Reovirus	Avelumab, paclitaxel	i.v.
NCT06660810	Soft tissue sarcoma, sarcoma, soft tissue	2	Herpes simplex virus Type 1	Talimogene laherparepvec, radiation	i.t.
NCT03120624	Metastatic endometrial carcinoma	1	Vesicular stomatitis virus	Ruxolitinib phosphate, technetium Tc‐99m sodium pertechnetate	i.v.
NCT06866977	Pancreatic cancer	1,2	Oncolytic alphavirus	Chemotherapy	∖
NCT06346808	Pancreatic cancer	1	Not provided	Chemotherapy, camrelizumab	∖
NCT06196671	Pancreatic cancer	2	Oncolytic adenovirus	Camrelizumab	i.t.
NCT06713148	Advanced malignant solid tumor	1	IDV‐SAFETM	∖	i.t.
NCT05361954	Cancer of pancreas, sarcoma, hepatic metastasis	1	Herpes simplex virus Type 1	∖	∖
NCT07211659	Ovarian cancer	1	Oncolytic adenovirus	∖	i.p.
NCT07170592	Treatment‐refractory solid tumors	1	Oncolytic vaccinia virus	Hydroxyurea, atezolizumab	i.t.
NCT07190833	Treatment‐refractory solid tumors	1	Oncolytic vaccinia virus	Hydroxyurea, atezolizumab	i.t.
NCT06919848	Biliary tract cancer	2	Oncolytic adenovirus	Lenvatinib, toripalimab, Folfox chemotherapy	i.v.
NCT05954091	Solid tumor	1	Herpes simplex virus Type 2	∖	i.t.
NCT06887348	Melanoma, metastatic melanoma, hepatocellular carcinoma	∖	Herpes simplex virus Type 1	∖	∖
NCT06283121	Gastric cancer, metastatic	2	Oncolytic adenovirus	SOX regimen, toripalimab	i.p.
NCT06283303	Colorectal cancer metastatic	∖	Herpes simplex virus Type 1	Toripalimab, regorafenib	∖
NCT06283134	Colorectal cancer metastatic	1	Oncolytic adenovirus	Toripalimab, regorafenib	∖
NCT06765954	High‐risk prostate cancer	2	Oncolytic adenovirus	Radiation therapy	s.c.
NCT07076498	Glioma	1	Herpes simplex virus Type 1	∖	i.t.
NCT07218692	Renal cell carcinoma	2	Herpes simplex virus Type 1	Tivozanib	i.t.
NCT07126990	Glioma	1,2	Oncolytic adenovirus	∖	i.t.
NCT06763965	Recurrent high‐grade glioma	1,2	Oncolytic adenovirus	∖	i.t.
NCT06215846	Solid tumor	1	Oncolytic adenovirus	∖	i.t.
NCT07145047	Glioblastoma	1,2	Not provided	∖	i.t.

*Data sources*: ClinicalTrials.gov.

The trial‐phase analysis indicates that most studies are early‐phase, classified as Phase I or Phase I/II, with relatively few advancing to Phase II/III. These findings suggest that OV‐based therapies remain largely in the early‐to‐intermediate stages of clinical translation. The registered trials cover a broad spectrum of solid tumors, including glioblastoma, melanoma, head and neck cancers, pancreatic cancer, breast cancer, gastrointestinal tumors, and other advanced or refractory solid malignancies. Notably, many studies have focused on patient populations with limited responses to standard therapies or ICIs, highlighting the need to evaluate OVs in settings of substantial unmet clinical need. Variations in tumor indications across viral backbones align with the distinct biological characteristics and clinical deployment strategies of individual OV platforms. Combination strategies are a major focus of contemporary OV research, with over one‐third of the registered trials evaluating OVs alongside ICIs, chemotherapy, or radiotherapy. These combination studies, primarily conducted in Phase I or Phase I/II, are designed to assess safety, tolerability, and preliminary efficacy. The growing emphasis on combination regimens reflects a strategic shift toward integrating OV therapies within established treatment paradigms rather than relying on monotherapy alone.

In summary, analysis of data from ClinicalTrials.gov indicates that OV therapy development is marked by a rapidly expanding clinical trial landscape, a high proportion of ongoing studies, diverse viral backbones, broad tumor coverage, and the frequent incorporation of rational combination strategies. This evolving clinical profile provides a foundation for the further optimization of OV platforms, refinement of target indications, and systematic evaluation of combination regimens in late‐stage clinical development.

## 7 Conclusions and Prospects

Across diverse viral platforms, accumulating preclinical and clinical evidence demonstrates that OVs can trigger ICD, enhance antigen release and presentation, and partially reprogram the TME toward a more immune‐permissive state. These distinctive properties distinguish OVs from conventional cytotoxic agents and position them as locally acting immune modulators with potential systemic effects. Despite this biological potential, the clinical responses remain variable, highlighting a persistent disconnection between mechanistic promise and therapeutic consistency. This discrepancy underscores the need to move beyond virus‐centric perspectives and to consider the complex interplay between viral biology, tumor architecture, and host immunity.

The current limitations of OVT are largely driven by biological barriers that restrict delivery, intratumoral propagation, and immune amplification in vivo. Following systemic administration, viral particles are rapidly neutralized or cleared by antiviral defenses, resulting in limited bioavailability and reduced access to the disseminated tumor sites. In solid tumors, abnormal vascular architecture, dense stroma, elevated interstitial pressure, hypoxia, and metabolic stress collectively restrict viral spread and replication. Simultaneously, immunosuppressive networks within the TME attenuate innate immune sensing and prevent virus‐induced inflammation from evolving into durable adaptive immunity. Together, these barriers help explain why OVT often achieves localized tumor lysis without eliciting durable systemic control.

Addressing these challenges requires a shift from virus‐centric optimization toward integrated therapeutic platform design. The efficacy of OVT depends not only on the virus itself but also on how effectively it is delivered, protected, and supported within the tumor ecosystem. Advanced delivery strategies, including cell‐based carriers, EV‐based carriers, and nanomaterial‐assisted encapsulation, represent convergent approaches to evade immune clearance and improve biodistribution. Importantly, these systems do not simply modify pharmacokinetics but actively shape the spatial and immunological contexts of viral infection. Their clinical translation requires scalable manufacturing, reproducible formulations, predictable release kinetics, and rigorous safety evaluations.

In parallel, the field shifted decisively away from monotherapy development. The biological activity of OVs is context‐dependent and shaped by the immune, stromal, and metabolic landscapes of the TME. Therefore, rationally designed combination strategies have become central to therapeutic progress. Pairing OVs with ICIs, chemoradiotherapy, metabolic modulators, or microenvironment‐targeting agents offers a way to synchronize viral cytolysis with immune reactivation and structural remodeling of the tumor niche. However, the success of such combinations depends on the careful orchestration of treatment timing, dosing, and mechanistic synergy.

Looking forward, OVT is likely to evolve into a programmable, adaptable immunotherapeutic platform rather than a uniform treatment modality. Advances in viral engineering enable localized expression of immunomodulatory payloads, stromal‐modifying factors, and context‐responsive regulatory elements to optimize viral activity under hostile tumor conditions. Simultaneously, increasing the recognition of tumors and host heterogeneity necessitates biomarker‐based patient stratification to identify settings in which viral replication, immune activation, and combination partners can be optimally aligned. From a translational perspective, advancing toward broader clinical implementation depends on the convergence of optimized delivery, rational combination strategies, and precise patient selection. While early‐phase studies have established the feasibility and safety of multiple platforms, achieving durable clinical benefits requires standardized evaluation frameworks that integrate virological and immunological endpoints, alongside robust manufacturing and regulatory strategies capable of supporting increasingly complex therapeutic constructs. Ultimately, the future of OVT depends on the systematic integration of virology, immunology, materials science, and clinical oncology to deliver reproducible and sustained therapeutic outcomes.

## Author Contributions

S.J., Y.Z., and X.W. conducted all literature searches relevant to the article's topic and contributed to drafting and writing the manuscript. Z.Z. and Y.Z. assisted with data collection. T.Y. and R.X. created all figures and tables. L.X. and J.Z. edited the manuscript. L.Z. and S.Y. provided grammar and language editing of the final manuscript. X.W. is the corresponding author. The final version of the manuscript was read and approved by all of the authors.

## Ethics Statement

The authors have nothing to report.

## Conflicts of Interest

The authors declare no conflicts of interest.

## Funding Information

This work was supported by the National Natural Science Foundation of China (82360044), the “12345 Future Talent Training Plan” of Zunyi Medical University (Future Science and Technology Elite Talent Project, ZYSE‐2024‐04), and the Guizhou Provincial Natural Science Foundation (Qian Ke He Ji Chu‐ZK[2024]‐269).

## Data Availability

The authors have nothing to report.
